# Head-to-Tail Cyclization and D-Amino Acid Substitution Redesign the Biological Activities of a Naturally Occurring Amphibian Peptide

**DOI:** 10.3390/molecules31183240

**Published:** 2026-09-14

**Authors:** María Verónica Húmpola, Roque Spinelli, Ivan Sanchís, Milagros de Orellana, Fernando Albericio, Álvaro Sebastian Siano

**Affiliations:** 1 Laboratorio de Peptidos Bioactivos, Department of Organic Chemistry, Faculty of Biochemistry and Biological Sciences, National University of the Littoral, Ciudad Universitaria, Santa Fe 3000, Argentina; 2 National Scientific and Technical Research Council (CONICET), Ministry of Science, Technology and Innovation, Godoy Cruz 2290, Ciudad de Buenos Aires C1425FQB, Argentina; 3 School of Chemistry and Physics, University of KwaZulu-Natal, Durban 4001, South Africa; 4 Department of Inorganic and Organic Chemistry, University of Barcelona, 08028 Barcelona, Spain

**Keywords:** amphibian peptides, cyclic peptide, D-amino acids, cholinesterase inhibitors, SARS-CoV-2, main protease inhibitors

## Abstract

Peptide engineering has emerged as a powerful strategy to optimize naturally occurring peptides. Here, the amphibian skin peptide Hp-1891 from *Boana pulchella* was selected as a model scaffold to investigate the effects of two complementary engineering approaches, namely site-specific D-amino acid substitution and head-to-tail cyclization. A library of twelve analogues was synthesized by 9-fluorenylmethyloxycarbonyl (Fmoc)-based solid-phase peptide synthesis and evaluated for inhibitory activity against acetylcholinesterase (AChE), butyrylcholinesterase (BChE), and the severe acute respiratory syndrome coronavirus 2 (SARS-CoV-2) main protease (Mpro), together with antioxidant and hemolytic activities. Circular dichroism spectroscopy and molecular modeling were performed to investigate the structural basis of the observed biological effects. Head-to-tail cyclization consistently enhanced inhibition of AChE, BChE, and Mpro, whereas D-amino acid substitution exerted a greater influence on antioxidant activity and hemolysis. Among the analogue library, c-Hp-d2 emerged as the most promising multifunctional peptide, displaying enhanced inhibition of all three enzymes while maintaining reduced hemolytic activity compared with the native peptide. Structural analyses indicated that cyclization promoted conformational organization, whereas D-amino acid incorporation reduced α-helical propensity. These findings demonstrate that rational peptide engineering effectively reshapes the biological profile of amphibian peptides and highlight head-to-tail cyclization as a versatile strategy for generating multifunctional peptide scaffolds with therapeutic potential.

## 1. Introduction

Peptides have emerged as one of the most promising classes of therapeutic molecules in modern drug discovery owing to their high target specificity, potent biological activity, favorable safety profiles, and ability to modulate protein–protein and protein–enzyme interactions that are often difficult to access with conventional small molecules [1,2,3]. These features have supported the development of an increasing number of peptide-based therapeutics for infectious diseases, cancer, metabolic disorders, and neurodegenerative conditions [1]. However, the value of peptides extends beyond their intrinsic biological activities. Naturally occurring peptides are increasingly viewed as privileged molecular scaffolds that can be rationally modified to produce analogues with improved pharmacological properties or even new biological functions. Consequently, a central challenge in peptide research is not only to discover new natural sequences, but also to determine how existing peptide structures can be redesigned to broaden their functional profile and therapeutic potential.

This paradigm has given rise to peptide engineering, in which naturally evolved sequences are used as starting templates for molecular optimization rather than as final therapeutic products. Progress in this area has been driven by advances in solid-phase peptide synthesis (SPPS), particularly Fmoc-based chemistry, which enables the efficient preparation of peptide analogues while allowing the precise incorporation of noncanonical amino acids and diverse structural modifications [4]. Thus, molecular features such as stereochemistry, conformational flexibility, amphipathicity, hydrophobicity, and charge distribution can be systematically adjusted to examine how specific structural changes influence biological function [5,6]. Beyond overcoming the inherent limitations of native peptides, these approaches offer a route to diversify biological activity and convert natural peptide scaffolds into multifunctional molecules.

Among the engineering strategies currently available, D-amino acid substitution and peptide cyclization are two of the most established approaches for peptide optimization. The incorporation of D-amino acids increases resistance to proteolytic degradation because endogenous proteases exhibit strict stereochemical specificity, while also affecting peptide conformation, membrane interactions, and target recognition [7]. Peptide cyclization, in turn, reduces conformational flexibility by preorganizing the molecule into bioactive conformations, thereby often improving target affinity, biological potency, and resistance to proteolytic degradation [8]. Importantly, these two strategies can produce distinct and complementary effects on peptide structure and biological activity, making their comparative evaluation particularly useful for defining peptide structure–activity relationships. Despite their broad application, relatively few studies have systematically investigated how these complementary engineering approaches reshape the functional landscape of naturally occurring peptide scaffolds [9].

Among natural peptide sources, amphibian skin secretions represent one of the richest reservoirs of structurally diverse bioactive molecules [10]. Produced by specialized granular glands as part of the innate immune defense system, these peptides have been reported to exhibit antimicrobial, antiviral, anticancer, immunomodulatory, antioxidant, and enzyme-inhibitory activities [11,12]. This functional diversity makes amphibian peptides attractive templates for rational peptide engineering. In previous work, our group identified and characterized Hp-1891, a 19-residue cationic α-helical peptide isolated from the skin secretion of Boana pulchellus [13]. Hp-1891 displayed potent antimicrobial activity but also showed significant hemolytic and cytotoxic effects associated with its pronounced hydrophobicity, limiting its direct therapeutic applicability. Nevertheless, these properties make Hp-1891 a useful scaffold for examining how rational structural modifications can redirect the biological behavior of a naturally occurring peptide.

In the present study, Hp-1891 was selected as a model scaffold to evaluate how two complementary peptide engineering strategies reshape the biological profile of a naturally occurring amphibian peptide. Novel analogues were generated through D-amino acid substitution and peptide cyclization using Fmoc-based SPPS and then evaluated across multiple biologically relevant activities. Their inhibitory potential against acetylcholinesterase (AChE) and butyrylcholinesterase (BChE), key enzymes involved in the cholinergic dysfunction associated with Alzheimer’s disease [11], and against the SARS-CoV-2 main protease (Mpro) [14], an essential enzyme for viral replication, was determined together with their antioxidant capacity. By comparing two classical peptide engineering strategies within the same natural scaffold, this work provides new insight into how rational chemical redesign can diversify peptide activity profiles and supports their development as multifunctional therapeutic candidates.

## 2. Results and Discussion

### 2.1. Rational Design of Hp-1891 Analogues

**Figure 1:**
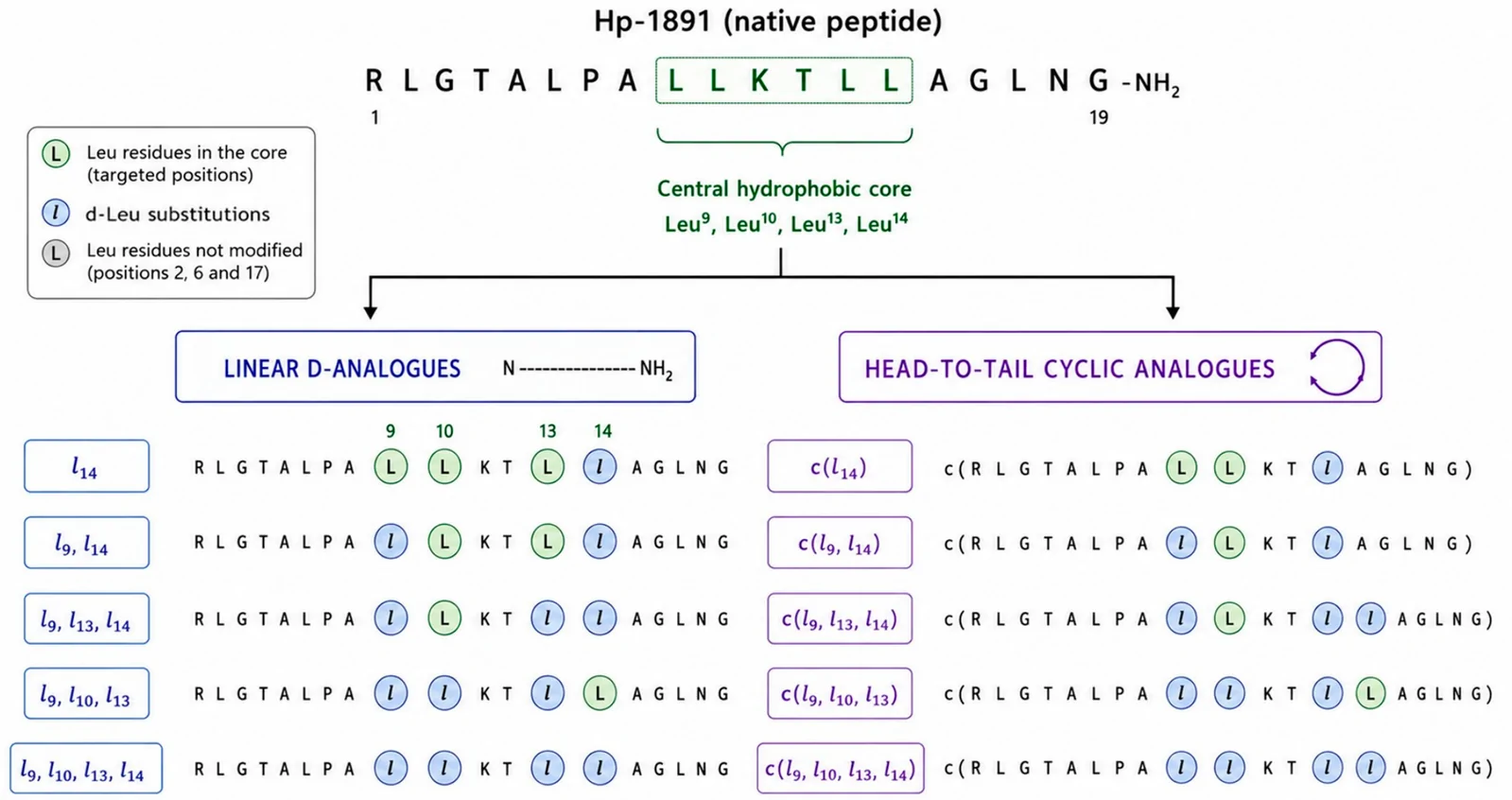
Schematic representation of the rational engineering strategy used to generate Hp-1891 peptide analogues. The native peptide was modified by site-specific D-leucine substitutions at residues Leu9, Leu10, Leu13, and Leu14 to produce five linear derivatives. The native peptide and each linear analogue were subsequently cyclized through head-to-tail cyclization, generating a library of 12 peptides for biological evaluation.

The design strategy was based on the previously characterized peptide Hp-1891 (H-RLGTALPALLKTLLAGLNG-NH_2_) from the skin secretion of *B. pulchellus*. Two complementary peptide engineering strategies were used: site-specific D-amino acid substitution and head-to-tail cyclization. Stereochemical inversion was restricted to the central hydrophobic region of Hp-1891, specifically at residues Leu9, Leu10, Leu13, and Leu14. These residues were selected because they form a contiguous hydrophobic cluster within the predicted amphipathic α-helix and were expected to play a major role in peptide conformation, membrane interactions, and biological activity. In contrast, the remaining leucine residues (Leu2, Leu6, and Leu17) were intentionally preserved to maintain the overall structural identity of the native scaffold. A detailed scheme of the design strategy is presented in Figure 1.

A progressive design strategy was adopted to evaluate the effect of increasing levels of stereochemical modification on peptide function. Five linear analogues were generated, comprising one peptide containing a single D-Leu substitution, one analogue with two D-Leu substitutions, two analogues with three D-Leu substitutions, and one analogue in which all four leucine residues within the central hydrophobic core were replaced by their D-enantiomers. This series enabled a systematic comparison of how the extent and distribution of stereochemical inversion influence the biological behavior of the same peptide scaffold.

To further investigate the influence of conformational restriction, each linear analogue was also converted into its corresponding head-to-tail cyclic peptide, yielding a second series of cyclic analogues. Cyclization was selected because it is one of the most widely employed strategies for peptide stabilization, reducing conformational flexibility while frequently enhancing resistance to proteolytic degradation, target affinity, and biological potency through the stabilization of bioactive conformations.

Overall, a total of 12 peptides were designed and synthesized from the native Hp-1891 scaffold. The library comprised the original linear peptide and its head-to-tail cyclic counterpart, as well as five D-amino acid-substituted analogues and their corresponding cyclic derivatives. This experimental design enabled a systematic comparison of the individual and combined effects of stereochemical inversion and backbone cyclization on the biological properties of the Hp-1891 scaffold.

### 2.2. Structural and Physicochemical Analysis

All peptides preserve the same order of 19 side-chain identities; the engineered changes are the configuration of selected Leu stereocenters and/or conversion of the C-terminally amidated linear backbone into a head-to-tail cyclic backbone. Accordingly, composition-based descriptors were invariant across the library: grand average of hydropathicity (GRAVY) was 0.837, the hydrophobic-residue fraction was 0.526, the aliphatic index was 159.5, and aromaticity was 0.000. These equal values do not imply structural equivalence, because L→D inversion changes backbone stereochemistry and side-chain presentation without changing the chemical identity or intrinsic hydropathy of the Leu side chain [15,16].

The topology-sensitive descriptors and experimental reverse-phase high-performance liquid chromatography (RP-HPLC) xretention times are summarized together in Table 1. Cyclization reduced the estimated net charge at pH 7.4 from +2.989 to +1.999 by removing the free N terminus, and changed the molecular weight from 1891.339 to 1874.308 Da, topological polar surface area (TPSA) from 755.59 to 715.58 Å^2^, hydrogen bond donors/acceptors (HBD/HBA) from 26/25 to 25/24, the formal RDKit rotatable-bond count from 62 to 26, and MolLogP from −6.663 to −5.977. The large change in rotatable-bond count is expected for macrocyclization because the standard topological definition excludes bonds that belong to rings; consequently, many backbone bonds counted in the linear chain no longer contribute after head-to-tail closure. This value is useful as a connectivity descriptor but does not quantify the actual torsional flexibility of the macrocycle. In parallel, the cyclic series showed substantially longer RP-HPLC retention times (20.75–23.60 min) than the linear series (4.63–6.24 min). Because residue composition is unchanged, this chromatographic shift is consistent with the combined effects of altered terminal charge, backbone topology and conformational presentation; retention time remains method-dependent and should not be interpreted as an experimental log*p* value.

**Figure 2:**
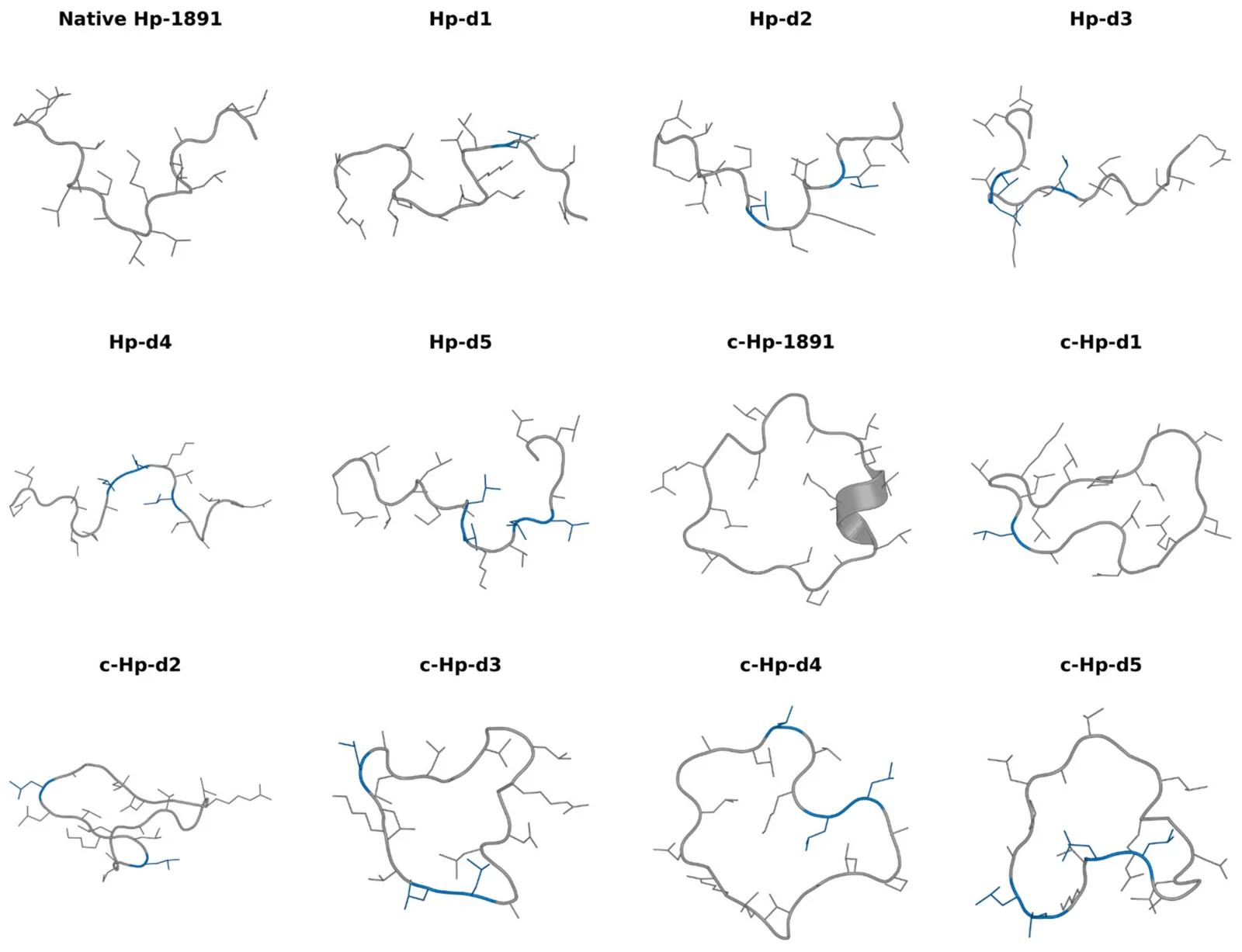
Representative aqueous structures of Hp-1891 and its linear and head-to-tail cyclic D-Leu analogues after Rosetta conformational sampling, explicit-water MD refinement and clustering. D-Leu residues are highlighted in blue.

The representative aqueous conformations provide the complementary three-dimensional view (Figure 2). Native Hp-1891 is predominantly extended, whereas the D-Leu analogues display position-dependent changes in backbone direction and compactness rather than a progressive structural change with increasing D-Leu content. Head-to-tail closure produces the expected macrocyclic topology, but the cyclic series remains conformationally heterogeneous. Several Leu and Ala side chains remain solvent-exposed around the rings and are therefore available for protein recognition. This behavior is consistent with the general observation that macrocyclization restricts the accessible conformational ensemble without necessarily producing a single rigid structure [17]. The aqueous structures also agree qualitatively with the CD data discussed below: the models retain substantial turn/coil character, while c-Hp-1891 contains a localized ordered segment compatible with its greater structural organization in water.

PeptiVerse web application (ChatterjeeLab, Hugging Face; SMILES input mode), using the main web version available on 28 July 2026 at 09:50 ART (UTC−3). PeptiVerse does not provide a numbered software release in the web interface. The corresponding PeptiVerse publication is cited as (10.1038/s41467-026-74167-w). PeptiVerse. Available online: https://huggingface.co/spaces/ChatterjeeLab/PeptiVerse (accessed on 28 July 2026).predictions revealed both topology- and stereochemistry-dependent differences in permeability-related parameters (Table 1; [18]). Native Hp-1891 and the partially substituted analogues Hp-d1, Hp-d2, Hp-d3, and Hp-d4 exhibited identical penetrance scores (0.68) and PAMPA permeability values (−12.702 log Peff). In contrast, the fully substituted analogue Hp-d5 showed a slightly lower penetrance score (0.658) and a more negative PAMPA permeability value (−13.276 log Peff). Thus, progressive D-Leu incorporation within the linear series did not substantially alter the predicted permeability-related parameters, with a detectable change emerging only when reaching the maximum substitution of four D-Leu residues.

Cyclization produced a distinct prediction profile. All six cyclic peptides had a penetrance score of 0.775 and a parallel artificial membrane permeability assay (PAMPA)value of −10.354 log Peff, corresponding to more favorable permeability-related predictions than the linear series, although PeptiVerse still classified all peptides as non-permeable in the PAMPA task [9,18].

### 2.3. Cholinesterase Inhibitory Activity

Cholinesterases (AChE and BChE) are key enzymes responsible for acetylcholine hydrolysis in the central nervous system and constitute validated therapeutic targets for the symptomatic treatment of Alzheimer’s disease [19]. While AChE predominates in the healthy brain, BChE activity increases during disease progression, making simultaneous inhibition of both enzymes an attractive strategy for maintaining cholinergic neurotransmission [11]. Since peptide cyclization and D-amino acid substitution can alter peptide conformation and target recognition, the inhibitory activities of the Hp-1891 analogue library against AChE and BChE were systematically evaluated.

**Figure 3:**
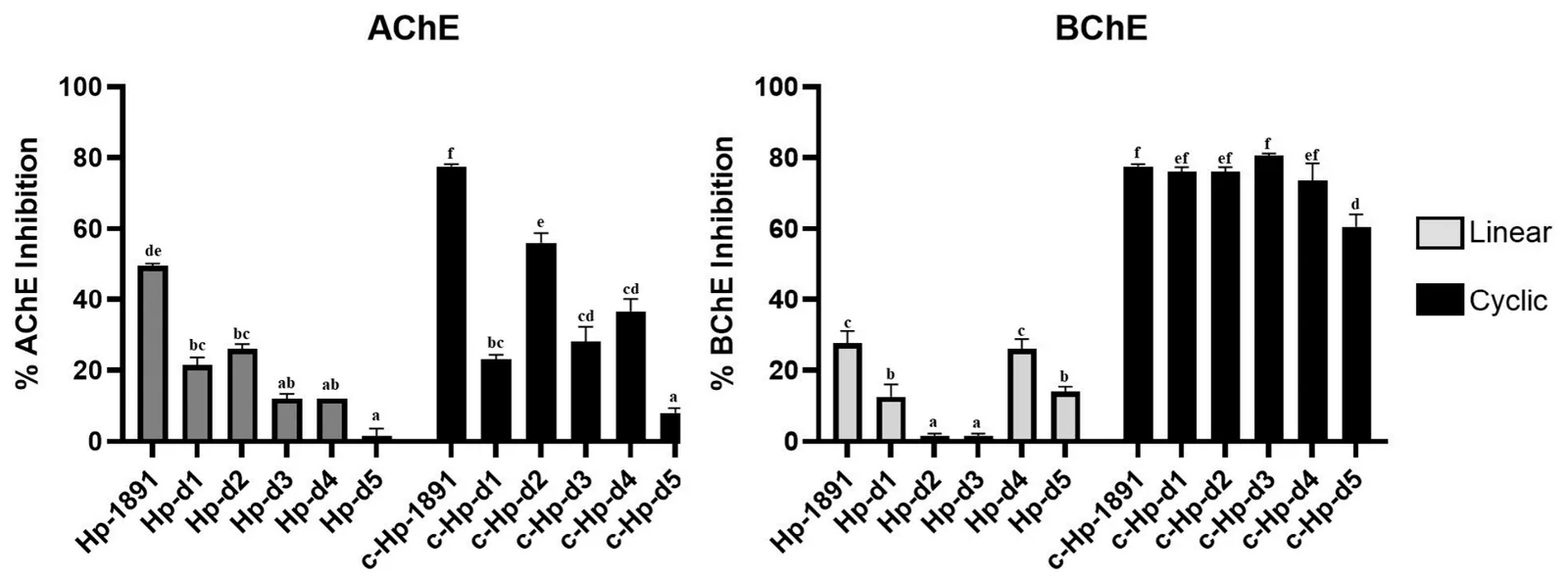
AChE and BChE inhibitory percentage of the linear and head-to-tail cyclic Hp-1891 analogues evaluated at a peptide concentration of 400 μM. Data are presented as mean ± standard error of the mean (SEM) (*n* = 3). Statistical significance was assessed independently for each enzyme by one-way ANOVA followed by Tukey’s HSD post hoc test (α = 0.05). AChE: F(11,12) = 181.97, *p* < 0.0001, HSD = 9.20; BChE: F(11,12) = 328.56, *p* < 0.0001, HSD = 10.12. Different letters above bars indicate statistically significant differences between groups (*p* < 0.05); groups sharing the same letter are not significantly different.

First, the inhibitory activities were evaluated at a fixed peptide concentration of 400 μM to assess their potential (Figure 3). This preliminary screening revealed distinct effects of the two engineering strategies. Whereas site-specific D-leucine substitutions alone produced only modest and position-dependent changes in cholinesterase inhibition, cyclization enhanced the inhibitory activity of the Hp-1891 scaffold against both enzymes.

For AChE, the native linear peptide Hp-1891 inhibited approximately 50% of the enzyme activity. In contrast, all linear D-substituted analogues exhibited inhibition below 30%, with Hp-d5 displaying almost no inhibitory activity. These results indicate that stereochemical inversion alone does not improve AChE inhibition and, depending on the substitution pattern, may even reduce enzyme inhibition. Remarkably, cyclization markedly enhanced inhibitory activity, with the cyclic native peptide (c-Hp-1891) reaching nearly 80% inhibition and representing the most active analogue in the library. Among the modified peptides, c-Hp-d2 exhibited the highest AChE inhibition, whereas the remaining cyclic analogues also displayed higher activities than their respective linear counterparts.

A similar behavior was observed for BChE (see Figure 3), although the effect of cyclization was even more pronounced. Most linear analogues showed weak inhibitory activity, while all cyclic peptides produced substantial inhibition, generally ranging between 60 and 80%. Interestingly, unlike AChE, the inhibitory activities of the cyclic derivatives toward BChE were relatively similar, suggesting that enzyme recognition is mainly governed by the conformational constraint imposed by cyclization rather than by the specific distribution of D-leucine residues. Only the fully substituted analogue, c-Hp-d5, exhibited a noticeable reduction in activity compared with the other cyclic peptides. The different inhibitory profile observed for BChE is likely related to the intrinsic structural properties of this enzyme. In contrast to AChE, BChE possesses a larger and more flexible active-site gorge, resulting from the replacement of several aromatic residues with smaller aliphatic amino acids and creating a more spacious binding cavity. Consequently, BChE displays well-recognized substrate and ligand promiscuity, accommodating structurally diverse molecules more readily than AChE [20,21]. This structural plasticity may explain why most cyclic analogues exhibited comparable inhibitory activities despite differences in the number and distribution of D-leucine substitutions. Conversely, the narrower and more restrictive active-site gorge of AChE appears to impose stricter structural requirements for ligand recognition, making its inhibition more sensitive to subtle stereochemical and conformational changes introduced into the Hp-1891 scaffold.

Overall, these findings demonstrate that cyclization is the dominant structural determinant governing cholinesterase inhibition within the Hp-1891 scaffold, whereas D-amino acid substitutions exert more subtle position-dependent effects by modulating peptide recognition rather than driving enzyme inhibition. The consistent enhancement observed after cyclization suggests that conformational restriction favors productive interactions with both cholinesterases, highlighting backbone cyclization as an effective strategy for transforming the natural antimicrobial peptide Hp-1891 into a dual cholinesterase inhibitor. Notably, among the cyclic analogues, there was no relationship between the number of D-Leu substitutions and cholinesterase inhibitory potency, since several cyclic analogues with intermediate D-Leu content outperformed both the unsubstituted c-Hp-1891 and the fully substituted c-Hp-d5. This indicates that D-Leu substitution fine-tunes the geometry of the cyclic peptide surface rather than acting as a uniformly activity-enhancing modification.

To further quantify the inhibitory potency of the most active analogues, dose–response assays were performed and IC_50_ values were determined (Figure S2 and Table 2). The dose–response analyses confirmed the trends observed in the initial screening. Based on the single-concentration screening results, dose–response analysis for AChE was restricted to the two most active cyclic analogues (c-Hp-1891 and c-Hp-d2), whereas all six cyclic peptides were carried forward to dose–response analysis for BChE given their broadly comparable activity in the initial screen. For AChE, c-Hp-1891 was the most potent inhibitor (IC_50_ = 91.5 μM), whereas c-Hp-d2 displayed a substantially higher IC_50_ value (310.6 μM). In contrast, all cyclic analogues exhibited measurable inhibitory activity against BChE, with IC_50_ values ranging from 45.5 to 149.1 μM. The lowest IC_50_ values were obtained for c-Hp-d1 (45.5 μM), c-Hp-d4 (49.3 μM), and c-Hp-d2 (54.4 μM), confirming that cyclization enhanced inhibitory potency toward BChE.

To gain structural insight into the enhanced inhibitory activity observed for the cyclic analogues, molecular dynamics (MD) simulations were performed using the most active peptide–enzyme complexes. Because a single scoring function is not a sufficient basis for selecting a peptide binding mode, all High Ambiguity Driven protein-protein DOCKing (HADDOCK)cluster representatives of every complex were first rescored with PRODIGY [22,23], a contact-based predictor of binding affinity for protein–protein and protein–peptide interfaces that estimates ΔG from interfacial contacts and non-interacting surface composition and therefore shares no term with the HADDOCK score. In all six systems, the pose promoted to MD ranked among the five most favourable poses of this independent ranking, and in two of them it was the top-ranked pose (Table S1), so the selected binding modes were retained for refinement.

Each complex was then simulated in three independent 100 ns replicates started from the same refined pose with independently generated initial velocities. All six receptors remained structurally stable, with mean backbone root mean square deviation (RMSD) ranging from 1.11 ± 0.06 Å for c-Hp-1891/TcAChE to 1.65 ± 0.07 Å for Hp-1891 linear/Mpro and no frame of any replicate exceeding 2.65 Å (Figure S4). The bound peptides fluctuated over a much wider range and clearly discriminated between the two backbone topologies (Figure S5). Across all three targets, the linear parent peptide departed further from its starting pose than the corresponding cyclic analogue (mean peptide RMSD over 50–100 ns, 6.75 ± 2.76 Å vs. 3.77 ± 2.07 Å; Mann–Whitney *p* = 0.013), with a consistently larger positional drift (4.63 ± 3.09 vs. 1.59 ± 1.80 Å per 100 ns; *p* = 0.022). Cyclization therefore stabilizes the bound state and not only the free peptide, and the representative structures analyzed below correspond to persistent binding modes rather than to transient poses.

### 2.4. Predicted Interactions with AChE

To investigate the molecular basis of the inhibitory profiles, the most active cyclic peptide for each enzyme was examined after HADDOCK docking and explicit-solvent MD refinement, using native linear Hp-1891 as the structural reference. Clustering and contact analyses were performed with ShineMD [24] over the last 20 ns of each of the three replicates of a complex, and the medoid of the most populated cluster was used for detailed structural views. Residue-pair occupancy maps report the percentage of analyzed frames in which each receptor-peptide pair formed a contact, so that interactions maintained across the three independent trajectories are distinguished from contacts present only in a representative snapshot. For AChE, comparison of Hp-1891 with c-Hp-1891 isolates the effect of cyclization because both peptides contain the same all-L amino acid sequence.

**Figure 4:**
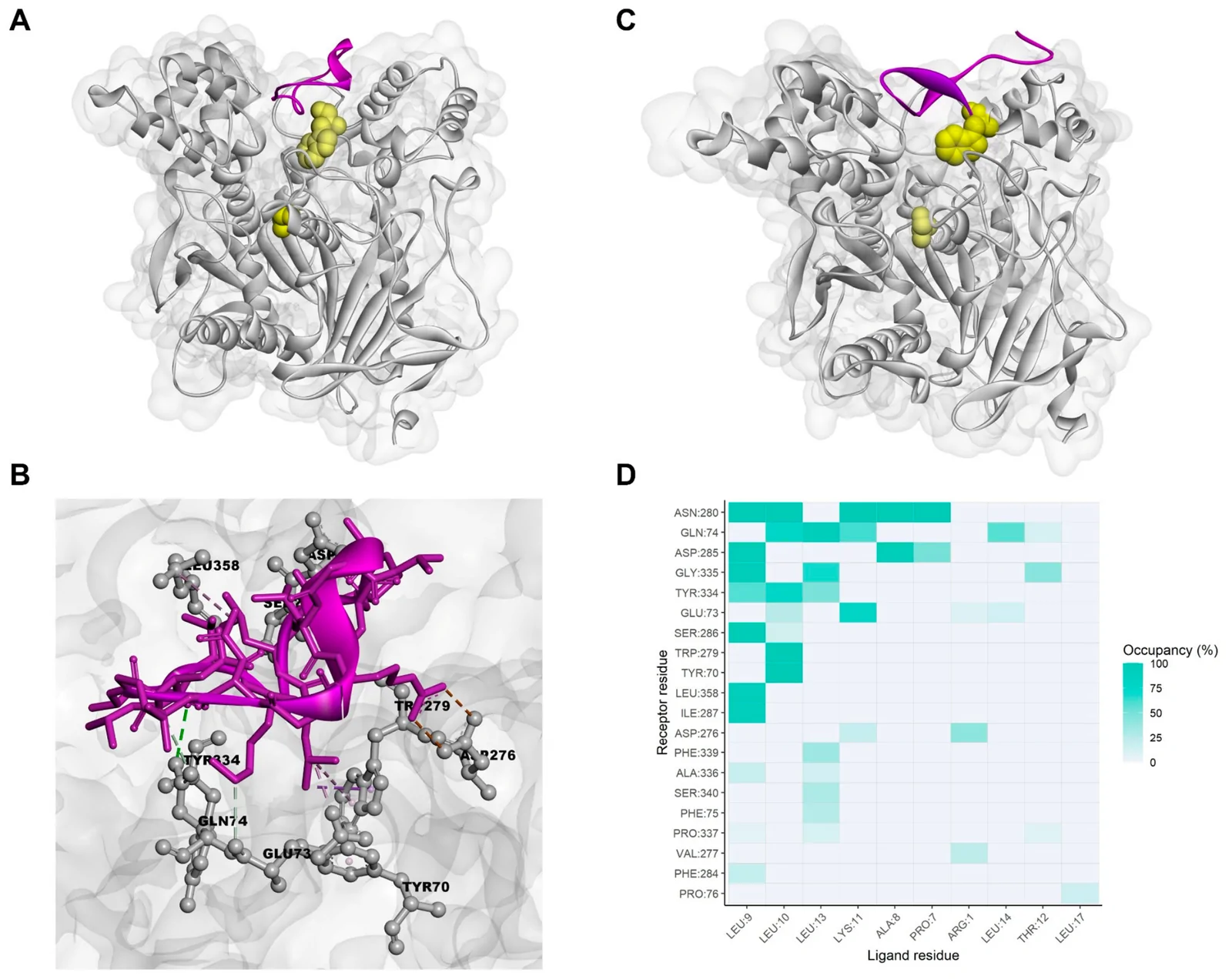
MD-refined interaction of Hp-1891 analogues with TcAChE. (**A**) Global position of linear Hp-1891 and (**C**) global position of c-Hp-1891 relative to the active-site gorge; yellow spheres indicate peripheral Trp279 and catalytic Ser200, used to define the insertion axis. (**B**) Detailed view of the cyclic peptide in the representative medoid structure obtained by clustering the last 20 ns of the three replicates pooled. (**D**) Receptor-peptide residue contact occupancy map calculated over the same pooled interval with ShineMD The version corresponding to Git commit b43d2893f801fcaceb29e779e0c18dd6ad54dc96 was used on 27 July 2026 at 15:22:37 ART (UTC−3). ShineMD is available at https://github.com/sanchisivan/ShineMD (accessed on 27 July 2026).

The *Torpedo californica acetylcholinesterase* (TcAChE)system shows that improved inhibition does not require extensive penetration toward the catalytic serine. The buried peptide fraction increased only from 28.1% for Hp-1891 to 29.9% for c-Hp-1891, while projection of the peptide centroid along the Trp279-Ser200 axis changed from −9.61 to −8.28 Å, corresponding to an approximately 1.3 Å displacement toward the catalytic region (Figure 4A,C). Trp279 was selected as the peripheral reference because it is a principal component of the TcAChE peripheral anionic site at the gorge entrance, whereas Ser200 is the catalytic nucleophile at its base; this vector therefore follows the functional entrance-to-catalytic-center direction of the gorge.

The more informative effect is the organization of the peripheral interaction interface. In the representative cyclic complex (Figure 4B), c-Hp-1891 is surrounded by Trp279, Tyr70, and Tyr334, together with Gln74, Glu73, Asp276, Asp285, Ser286, Ile287, and Leu358. Trp279, Tyr70, and Tyr334 form part of the aromatic peripheral region involved in ligand recognition and control of access to the active-site gorge. The occupancy map (Figure 4D) shows persistent Trp279-Leu10 and Tyr70-Leu10 contacts and a highly occupied Ser286-Leu9 interaction; Leu9 also contacts Ile287 and Leu358, while Tyr334, Asn280, Gln74, and Glu73 maintain recurrent interactions with several peptide positions. Thus, cyclization generates a distributed and persistent peripheral interface rather than a single anchoring contact.

This binding region is consistent with previous peptide-AChE studies. In the Hp-1935-derived LL series, Pro3 contacted Tyr70, Trp279, and Tyr334, and replacement with Trp strengthened inhibition through the same peripheral region [25]. W3 and the unrelated peptide BcI-4 also engaged the analogous hAChE peripheral residues Asp74, Trp286, and Tyr341 [26,27]. c-Hp-1891 therefore reaches a recurrent peptide-recognition region through a different mechanism, with macrocyclization organizing multiple aliphatic side chains into a hydrophobic surface at the gorge entrance. This is compatible with peripheral inhibition by larger peptide ligands, as structurally exemplified by fasciculin [28].

### 2.5. Predicted Interactions with BChE

For BChE, the selected cyclic lead is c-Hp-d1. Comparison with native Hp-1891 therefore reflects the combined consequences of head-to-tail cyclization and the D-Leu14 substitution; the complete analogue series is used to distinguish the common effect of cyclization from stereochemistry-dependent tuning.

**Figure 5:**
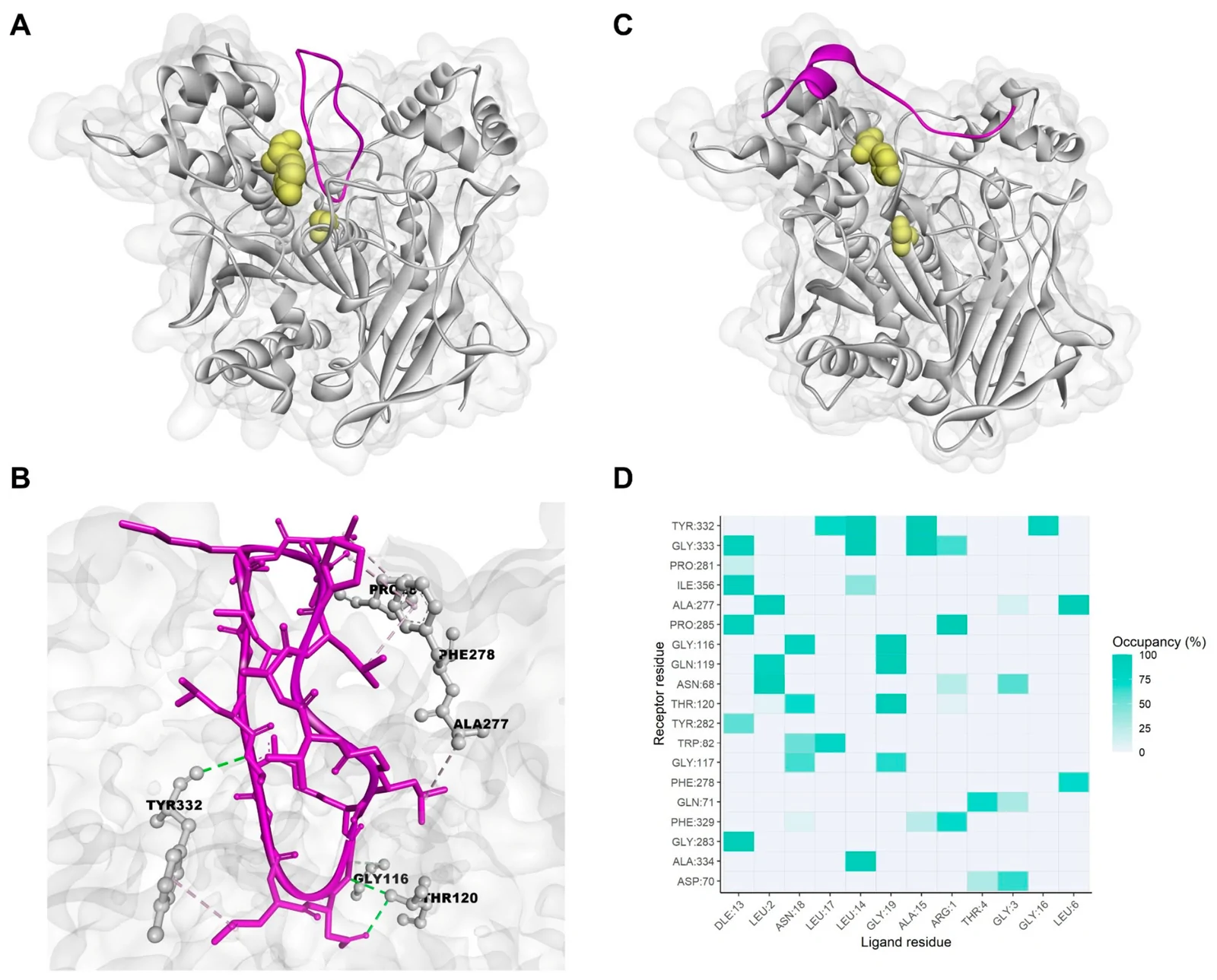
MD-refined interaction of Hp-1891 analogues with human BChE. (**A**) Global position of linear Hp-1891 and (**C**) global position of the BChE lead c-Hp-d1; yellow spheres indicate Tyr332 and catalytic Ser198, which define the gorge insertion axis. (**B**) Detailed interaction network of c-Hp-d1 in the representative medoid structure obtained by clustering the last 20 ns of the three replicates pooled. (**D**) Receptor-peptide residue contact occupancy map calculated over the same pooled interval with ShineMD.

A markedly different behavior was observed for hBChE. The buried peptide fraction increased from 31.1% for Hp-1891 to 45.5% for c-Hp-d1, and the centroid position along the Tyr332-Ser198 axis changed from -8.28 to +2.86 Å, corresponding to an approximately 11.1 Å displacement toward the catalytic region (Figure 5A,C). Tyr332 was selected as the peripheral reference because it contributes to ligand recognition near the mouth of the BChE gorge, whereas Ser198 is the catalytic serine; this axis therefore describes movement along the functional direction of the cavity. The much larger shift relative to AChE indicates genuine deeper accommodation of the cyclic lead.

This behavior is consistent with the broader and more flexible BChE gorge discussed above, which favors accommodation of bulkier ligands [20,21]. In the representative c-Hp-d1 complex (Figure 5B), the peptide is enclosed by Tyr332 together with Pro281, Phe278, Ala277, Gly116 and Thr120. The occupancy map (Figure 5D) extends this picture: Tyr332 and neighboring Gly333 maintain persistent contacts with the cyclic hydrophobic region, Ala277 strongly contacts Leu residues, Pro285 engages the D-Leu-containing region and Arg1, and Gly116, Gln119 and Thr120 form recurrent contacts with the C-terminal region. Contacts also extend toward Trp82, Phe329 and peripheral Asp70.

These residues span functionally distinct regions of BChE. Tyr332 and Asp70 contribute to peripheral recognition, Trp82 belongs to the choline-binding region, Phe329 lies in the aromatic/anionic region of the gorge, and Gly116/Gly117 are positioned closer to the catalytic side of the cavity. The persistent contact network therefore supports the insertion metric: c-Hp-d1 simultaneously engages peripheral and deeper recognition regions. Similar hotspots were identified previously for W3W7, in which Trp7 contacted Tyr332/Phe329, and Leu10 contacted Trp82 [26]. The fact that c-Hp-d1 reaches these receptor nodes without aromatic peptide residues highlights how macrocyclic preorganization can generate an alternative complementary hydrophobic surface. The complete experimental series nevertheless shows no monotonic relationship between D-Leu number and BChE potency, indicating that stereochemistry tunes the geometry of the cyclic surface rather than acting as an intrinsically activity-enhancing modification.

These findings demonstrate that rational peptide engineering substantially improved the cholinesterase inhibitory profile of Hp-1891. While the native peptide displayed only moderate inhibition, cyclization generated analogues with markedly enhanced potency against both AChE and BChE. In the context of current therapeutic strategies for Alzheimer’s disease, which increasingly emphasize multi-target modulation rather than single-enzyme inhibition, these results highlight the potential of rational peptide engineering to transform naturally occurring antimicrobial peptides into multifunctional scaffolds for neurodegenerative drug discovery. Although the cyclic analogues remain less potent than rivastigmine (IC_50_ = 33.17 μM for AChE and 0.08 μM for BChE), peptide-based inhibitors such as c-Hp-1891 and c-Hp-d1 offer structurally distinct modes of engagement, involving peripheral interface reorganization and deeper gorge accommodation, respectively, together with a favorable hemolytic profile for several analogues in the same library. This combination of dual-enzyme activity and tunable safety, rather than potency alone, supports the value of these scaffolds as starting points for further multi-target optimization against Alzheimer’s disease-relevant targets.

### 2.6. Mpro Inhibitory Activity

The severe acute respiratory syndrome coronavirus 2 (SARS-CoV-2) main protease (Mpro, also known as 3CLpro) is an essential cysteine protease responsible for processing viral polyproteins into the non-structural proteins required for viral replication and transcription. Owing to its indispensable role in the viral life cycle and the absence of closely related human homologues, Mpro has emerged as one of the most attractive molecular targets for antiviral drug development [29]. Since peptide cyclization and stereochemical modifications can influence target recognition by altering conformational flexibility, the inhibitory activity of the Hp-1891 analogue library against SARS-CoV-2 Mpro was evaluated.

**Figure 6:**
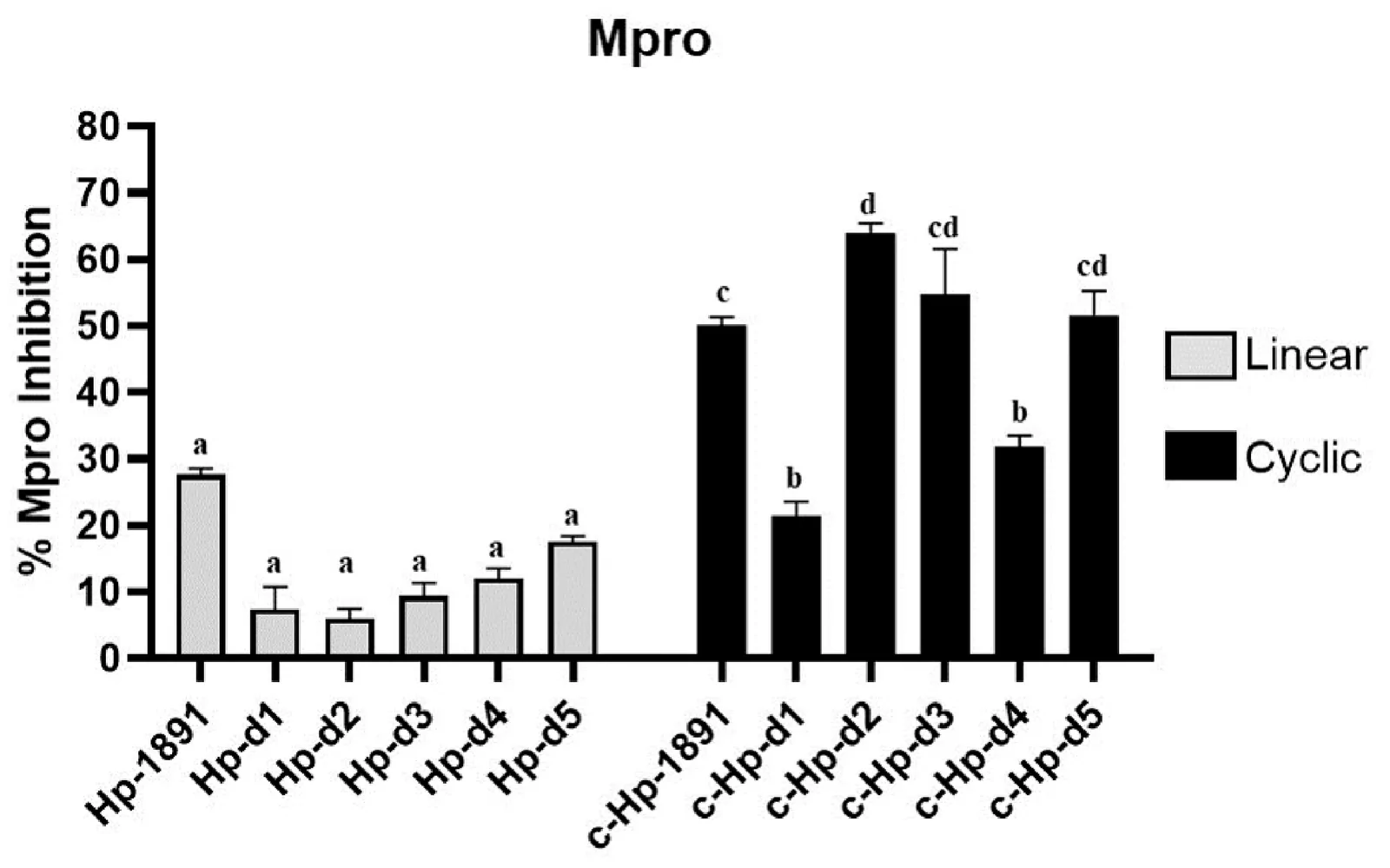
Mpro inhibitory percentage of the linear and head-to-tail cyclic Hp-1891 analogues evaluated at a peptide concentration of 800 μM. Data are presented as mean ± SEM (n = 3). Statistical significance was assessed by one-way ANOVA followed by Tukey’s HSD post hoc test (α = 0.05; F(11,12) = 118.22, *p* < 0.0001; HSD = 11.14). Different letters above bars indicate statistically significant differences between groups (*p* < 0.05); groups sharing the same letter are not significantly different.

Figure 6 shows the inhibitory activities of the native peptide and its engineered analogues at a fixed peptide concentration of 800 μM. Similar to the behavior observed for cholinesterases, head-to-tail cyclization produced a marked enhancement of Mpro inhibition, whereas site-specific D-leucine substitutions alone resulted in only modest or negligible changes in activity. Among all analogues, c-Hp-d2 exhibited the highest inhibitory activity (63.0%), representing an approximately 9-fold increase compared with its linear counterpart Hp-d2 (7.1%) and more than a twofold improvement over the native peptide Hp-1891 (28.3%). Consistent with the preliminary screening, dose–response analysis confirmed that c-Hp-d2 was the most potent analogue within the peptide library, yielding an IC_50_ value of 332 μM (Table 3 and Figure S3) and further supporting cyclization as the principal contributor to the enhanced antiviral activity of the Hp-1891 scaffold.

In a previous study conducted in the context of the coronavirus disease 2019 (COVID-19) pandemic, our group demonstrated that amphibian skin secretions constitute a valuable source of natural Mpro inhibitors by screening 23 peptides and identifying Hp-1081 and Hp-1971 as the most active compounds, with IC_50_ values of 41.3 and 66.4 μM, respectively [14]. Unlike those naturally occurring peptides, the present work shows that rational chemical engineering through cyclization can markedly enhance the antiviral activity of a previously characterized peptide scaffold. Although c-Hp-d2 did not surpass the potency of the best natural inhibitors, the substantial improvement achieved relative to native Hp-1891 highlights cyclization as a promising strategy for optimizing amphibian-derived peptide scaffolds. These findings open new opportunities for the rational design of focused libraries of cyclic analogues with improved antiviral potential.

The improvement in Mpro inhibition observed after cyclization is consistent with previous reports describing the beneficial effects of peptide cyclization on viral protease inhibition. Zhang et al. engineered cyclic analogues of the conotoxin-derived peptide MrIA as inhibitors of the dengue virus NS2B-NS3 protease and demonstrated that backbone cyclization markedly increased inhibitory potency compared with the corresponding linear peptides [30]. The authors attributed this enhancement to the conformational constraint imposed by cyclization, which favored productive interactions with the catalytic pocket while also improving peptide stability against proteolytic degradation. These observations closely parallel the behavior of the Hp-1891 scaffold, supporting the concept that cyclization is an effective strategy for optimizing peptide inhibitors targeting viral proteases.

### 2.7. Predicted Interactions with Mpro

For Mpro, the selected cyclic lead was c-Hp-d2. Accordingly, comparison with native Hp-1891 captures the combined effects of macrocyclization and the D-Leu9/D-Leu14 substitution pattern, while the experimental series provides the broader structure–activity context.

**Figure 7:**
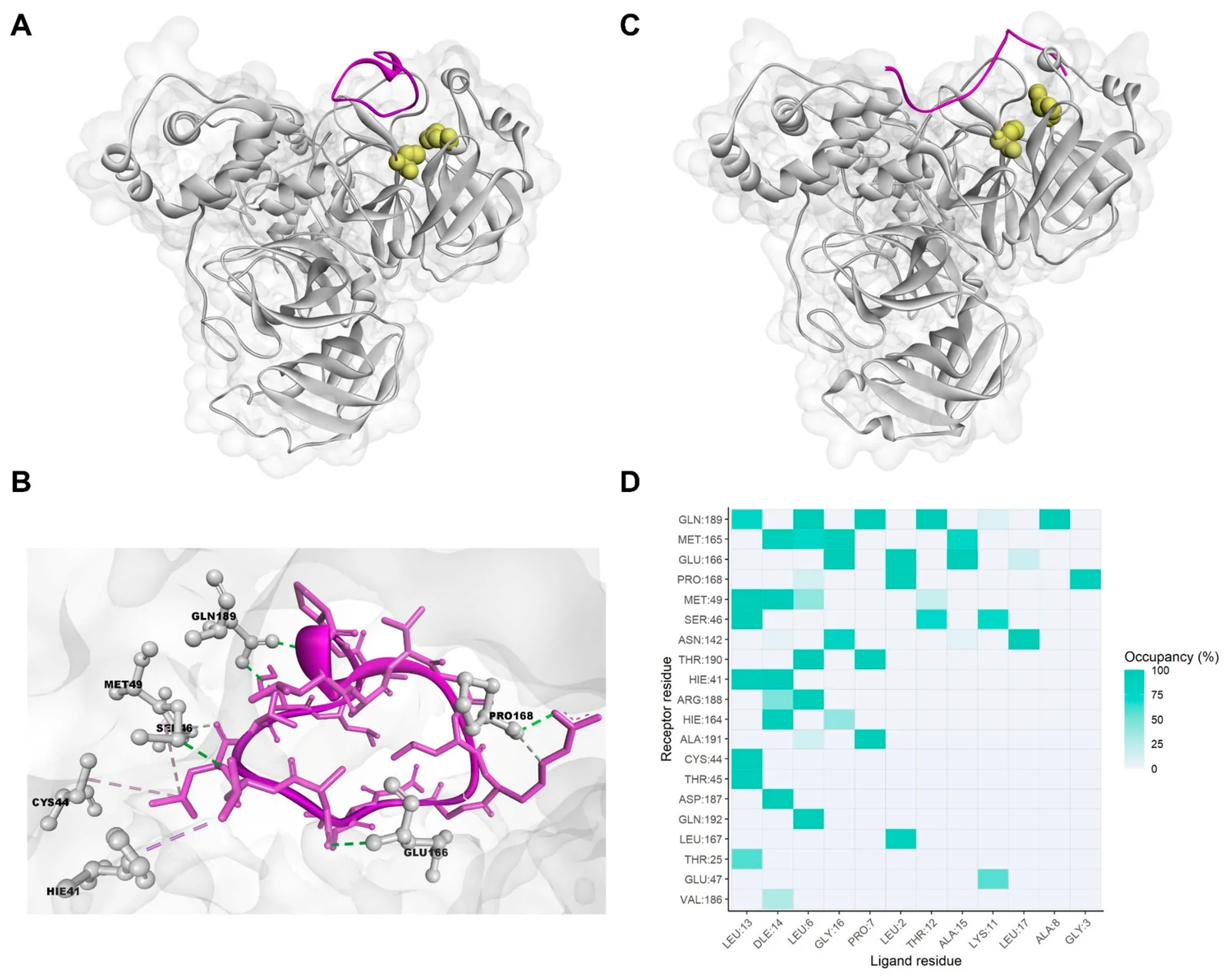
MD-refined interaction of Hp-1891 analogues with SARS-CoV-2 Mpro. (**A**) Global position of linear Hp-1891 and (**C**) global position of the Mpro lead c-Hp-d2 relative to the catalytic region; yellow spheres indicate His41 and Cys145, whose midpoint was used as the catalytic reference center. (**B**) Detailed interaction network of c-Hp-d2 in the representative medoid structure obtained by clustering the last 20 ns of the three replicates pooled. (**D**) Receptor-peptide residue contact occupancy map calculated over the same pooled interval with ShineMD.

Mpro presents a third and mechanistically distinct binding environment. Its substrate-recognition region is an open cleft organized around the His41-Cys145 catalytic dyad, so a one-dimensional insertion axis is not appropriate. Instead, occupation of the catalytic environment was quantified relative to the midpoint of His41 and Cys145. The buried fraction increased from 33.5% for Hp-1891 to 38.9% for c-Hp-d2, while the fraction of peptide heavy atoms within 10 Å of the catalytic center increased from 5.3% to 16.7%; five residues of the cyclic peptide occupied this region compared with three residues in the linear form (Figure 7A,C).

The representative c-Hp-d2 structure (Figure 7B) is positioned among His41, Cys44, Ser46, Met49, Glu166, Pro168, and Gln189. The occupancy map (Figure 7D) shows persistent contacts of the Leu13/D-Leu14 segment with His41 and the adjacent Cys44-Thr45-Ser46-Met49 region; D-Leu14 also contacts Met165. Glu166 interacts recurrently with several peptide positions, while Gln189 forms one of the broadest interaction hubs in the map. Asn142, Thr190, Arg188, and Ile164 provide additional contacts. His41/Cys145 form the catalytic dyad, whereas Glu166, Met165, Gln189, and Asn142 are established components of substrate and inhibitor recognition around the Mpro cleft [31].

The same Gln189/Glu166/Asn142 region was identified in our previous docking/MD analysis of the natural amphibian peptide Hp-1081 [14], supporting its relevance for peptide accommodation. c-Hp-d2 also maintains persistent contacts with catalytic His41, whereas Cys145 is not among the dominant occupancy pairs. The simulations therefore support a noncovalent mechanism based on occupation and obstruction of the substrate-recognition cleft rather than direct covalent engagement of Cys145. A benefit of cyclization for Mpro inhibition has also been reported for the cyclic substrate-mimetic UCI-1, whose linear controls showed little activity [32].

Across the three enzymes, cyclization is the common modification most consistently associated with enhanced inhibition, but its structural consequence is target-dependent: c-Hp-1891 primarily reorganizes a peripheral AChE interface, c-Hp-d1 is accommodated substantially deeper in the BChE gorge, and c-Hp-d2 increases occupation of the Mpro catalytic environment. The occupancy maps show that these binding modes are maintained by persistent interaction networks rather than by isolated snapshot contacts, while the different optimal stereochemical patterns reinforce that D-Leu substitution fine-tunes target recognition rather than providing a universal activity gain.

### 2.8. Antioxidant Activity

Oxidative stress is a common pathological hallmark of both neurodegenerative disorders and viral infections. In Alzheimer’s disease, excessive production of reactive oxygen species (ROS) contributes to neuronal damage, lipid peroxidation, protein oxidation, and amyloid-β toxicity, thereby accelerating disease progression [11]. Likewise, during SARS-CoV-2 infection, oxidative stress is closely associated with mitochondrial dysfunction, hyperinflammation, cytokine release and tissue injury, contributing to disease severity [33]. Consequently, compounds capable of inhibiting disease-related enzymes while scavenging free radicals are particularly attractive as multifunctional therapeutic agents. Therefore, the antioxidant activity of the Hp-1891 analogue library was evaluated using the 2,2′-diphenyl-1-picrylhydrazyl (DPPH) radical scavenging assay.

**Figure 8:**
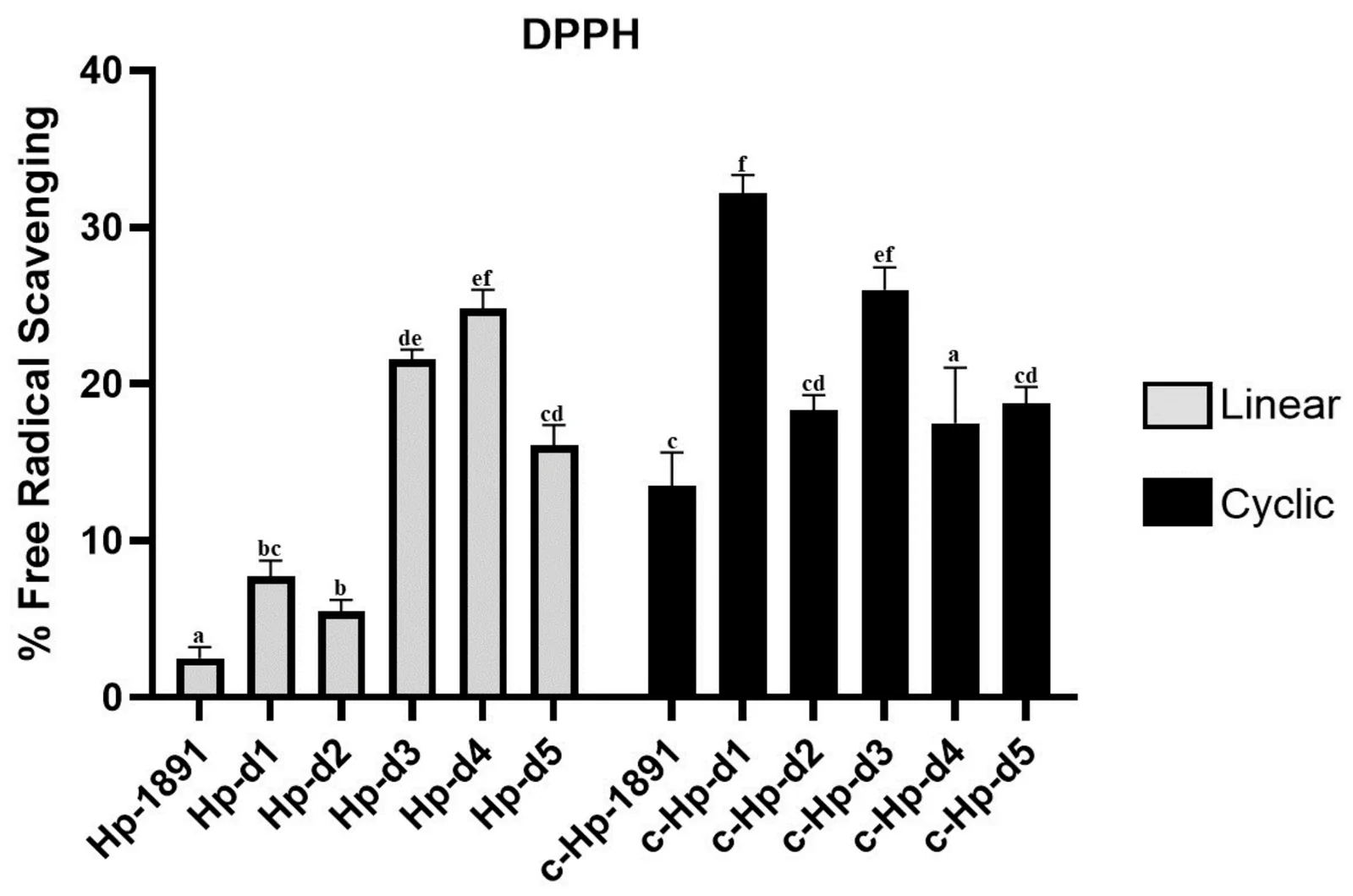
DPPH free radical scavenging percentage of the Hp-1891 analogues at a peptide concentration of 400 μM. Data are presented as mean ± SEM (n = 3). Statistical significance was assessed by one-way ANOVA followed by Tukey’s HSD post hoc test (α = 0.05; F(11,12) = 134.79, *p* < 0.0001; HSD = 4.78). Different letters above bars indicate statistically significant differences between groups (*p* < 0.05); groups sharing the same letter are not significantly different.

Overall, rational structural engineering improved the antioxidant capacity of the Hp-1891 scaffold. The native peptide exhibited only weak DPPH scavenging activity at a concentration of 400 μM (2.5%), whereas several modified analogues displayed markedly higher antioxidant activities (Figure 8). Among the linear derivatives, Hp-d3 and Hp-d4 showed the highest radical scavenging capacities, reaching approximately 21.6 and 24.8%, respectively, at the evaluated concentration. Head-to-tail cyclization further enhanced the antioxidant activity of several analogues, with c-Hp-d1 emerging as the most active peptide in the library (32.2%), followed by c-Hp-d3 (26.0%).

Amphibian skin is highly permeable and continuously exposed to ultraviolet radiation, atmospheric oxygen, and microbial pathogens, making oxidative stress an important physiological challenge. Consequently, amphibians have evolved an efficient antioxidant defense system that includes bioactive peptides capable of scavenging reactive oxygen species and protecting skin tissues from oxidative damage [34,35]. Peptide antioxidant activity is strongly influenced by structure–activity relationships, with hydrophobicity, molecular weight, amino acid composition, and residue distribution representing key determinants of radical-scavenging capacity [36]. The native peptide Hp-1891 displayed only negligible antioxidant activity, indicating that its natural sequence is not intrinsically optimized for free-radical scavenging.

Interestingly, peptide engineering substantially improved the antioxidant activity of several analogues. Previous studies have shown that the incorporation of D-amino acids can enhance antioxidant activity by modifying peptide conformation, increasing structural stability, and improving the accessibility and orientation of amino acid side chains involved in radical scavenging. Rather than acting through changes in amino acid composition, stereochemical inversion appears to optimize the spatial arrangement of antioxidant residues, facilitating more effective interactions with reactive oxygen species. This mechanism is consistent with the improved activities observed for several D-substituted Hp-1891 analogues, particularly Hp-d3 and Hp-d4 [37]. Cyclization further enhanced the antioxidant capacity of selected analogues. In addition to increasing conformational stability, cyclization has been reported to modify peptide hydrophobicity and residue exposure, properties that strongly influence antioxidant performance [38]. Together, these findings suggest that both stereochemical inversion and conformational restriction contribute to the improved radical-scavenging activity observed in the engineered Hp-1891 library, although their effects are clearly sequence-dependent.

Overall, the antioxidant results complement the cholinesterase and SARS-CoV-2 Mpro inhibition assays, further showing that rational structural engineering can expand the functional profile of the Hp-1891 scaffold.

### 2.9. Hemolytic Activity

One of the major limitations preventing the therapeutic application of many amphibian peptides is their cytotoxicity [12]. Owing to their highly amphipathic α-helical structures and elevated hydrophobicity, these peptides frequently exhibit strong hemolytic activity resulting from non-selective disruption of erythrocyte membranes. Since Hp-1891 was previously reported to be highly hemolytic, the effects of D-leucine substitution and head-to-tail cyclization were evaluated using human erythrocytes.

**Figure 9:**
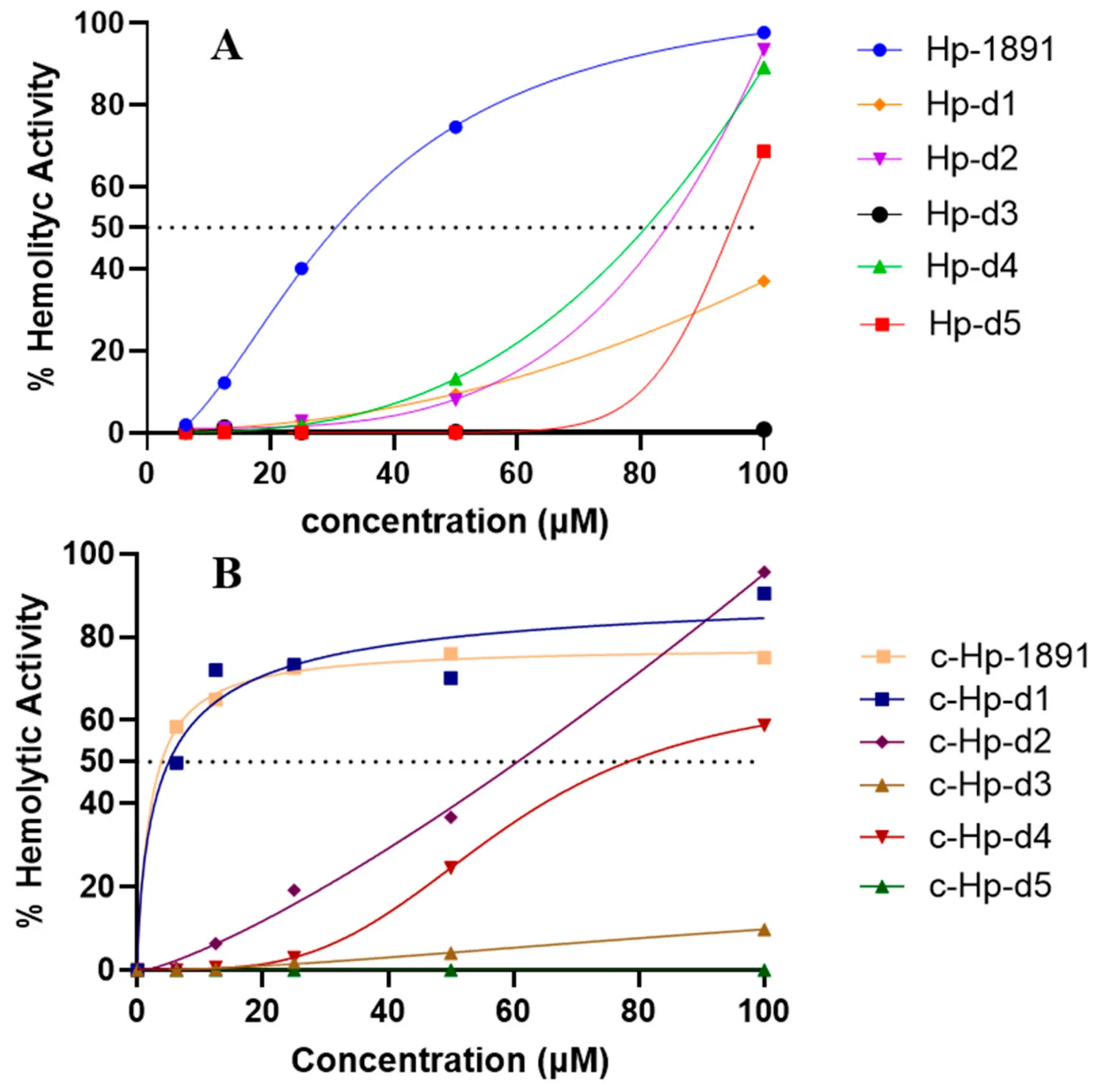
Dose–response curves of the hemolytic activity of Hp-1891 and its engineered analogues against human erythrocytes. (**A**) Linear series and (**B**) cyclic series.

The hemolytic activities of the native peptide and its analogues were determined over a concentration range of 6.25–100 μM (Figure 9). As expected, native Hp-1891 displayed pronounced hemolytic activity, causing nearly complete erythrocyte lysis at 100 μM.

Overall, site-specific D-leucine substitutions substantially reduced hemolysis across most analogs, although the magnitude of this effect strongly depended on the substitution pattern. Among the linear analogues, all showed a marked reduction in hemolysis compared with the native peptide at lower concentrations (6.25–50 μM). However, at the highest concentration tested (100 μM), Hp-d3 exhibited an almost complete loss of hemolytic activity, whereas Hp-d2, Hp-d4 and Hp-d5 reached moderate to high levels. This indicates that not all stereochemical substitutions affect membrane disruption to the same extent.

Head-to-tail cyclization alone did not consistently reduce hemolysis. The cyclic native peptide (c-Hp-1891) remained highly hemolytic, indicating that conformational restriction by itself is insufficient to improve membrane selectivity. Instead, the hemolytic behavior of the cyclic analogues was mainly determined by the number and position of the incorporated D-leucine residues. In general, an increased amount of D-Leu correlated with decreased hemolytic activity. Notably, c-Hp-d3 and c-Hp-d5 exhibited almost negligible hemolysis across the entire concentration range. For both linear and cyclic analogues, the greatest reduction in hemolytic activity was achieved through the simultaneous substitution of the L9, L13 and L14 residues.

The reduction in hemolytic activity observed after D-amino acid incorporation is consistent with previous studies showing that stereochemical inversion disrupts the continuous amphipathic α-helical arrangement required for efficient membrane permeabilization [39]. We previously reported that the incorporation of D-amino acids into antimicrobial peptides significantly decreased hemolytic activity while preserving or even improving selected biological properties, an effect attributed to reduced α-helical propensity and weaker interactions with zwitterionic mammalian membranes [40]. Our results further show that this effect is highly position-dependent, as substitutions at different leucine residues produced markedly distinct hemolytic profiles despite minimal changes in the primary sequence.

This modulation of hemolytic activity appears to be closely linked to changes in peptide hydrophobicity. Interestingly, the least hemolytic D-analogues correspond to the least hydrophobic members of each series, as shown by their HPLC retention times: Hp-d3 (tR = 4.63 min) for the linear peptides, and c-Hp-d3 (tR = 21.51 min) and c-Hp-d5 (tR= 20.75 min) for the cyclic ones. This indicates that both hydrophobicity and the α-helical arrangement are among the main parameters governing the interaction of these analogues with the erythrocyte membrane.

The correlation between the biological and hemolytic profiles of the synthesized library reveals a clear mechanistic decoupling of enzymatic inhibition from non-specific membrane disruption. Notably, pronounced hemolytic activity does not ensure biological efficacy, as evidenced by the highly toxic but enzymatically inactive analogues Hp-d2, Hp-d4, and c-Hp-d1. Conversely, while c-Hp-d2 emerges as the most potent multifunctional analogue, combining enhanced inhibition of AChE, BChE, and SARS-CoV-2 Mpro alongside measurable antioxidant activity, it exhibits elevated hemolytic activity at high concentrations. In contrast, the rationally designed analogues c-Hp-d3 and c-Hp-d5 successfully uncouple these effects. These latter peptides present an exceptional safety profile with negligible hemolysis, and despite a partial attenuation in efficacy, particularly against AChE, they preserve pharmacologically relevant inhibition of BChE and SARS-CoV-2 Mpro. Collectively, these findings demonstrate that rational peptide engineering enables the independent modulation of biological potency and membrane selectivity, highlighting the versatility of the Hp-1891 scaffold for the development of multifunctional peptide therapeutics.

### 2.10. Circular Dichroism

**Figure 10:**
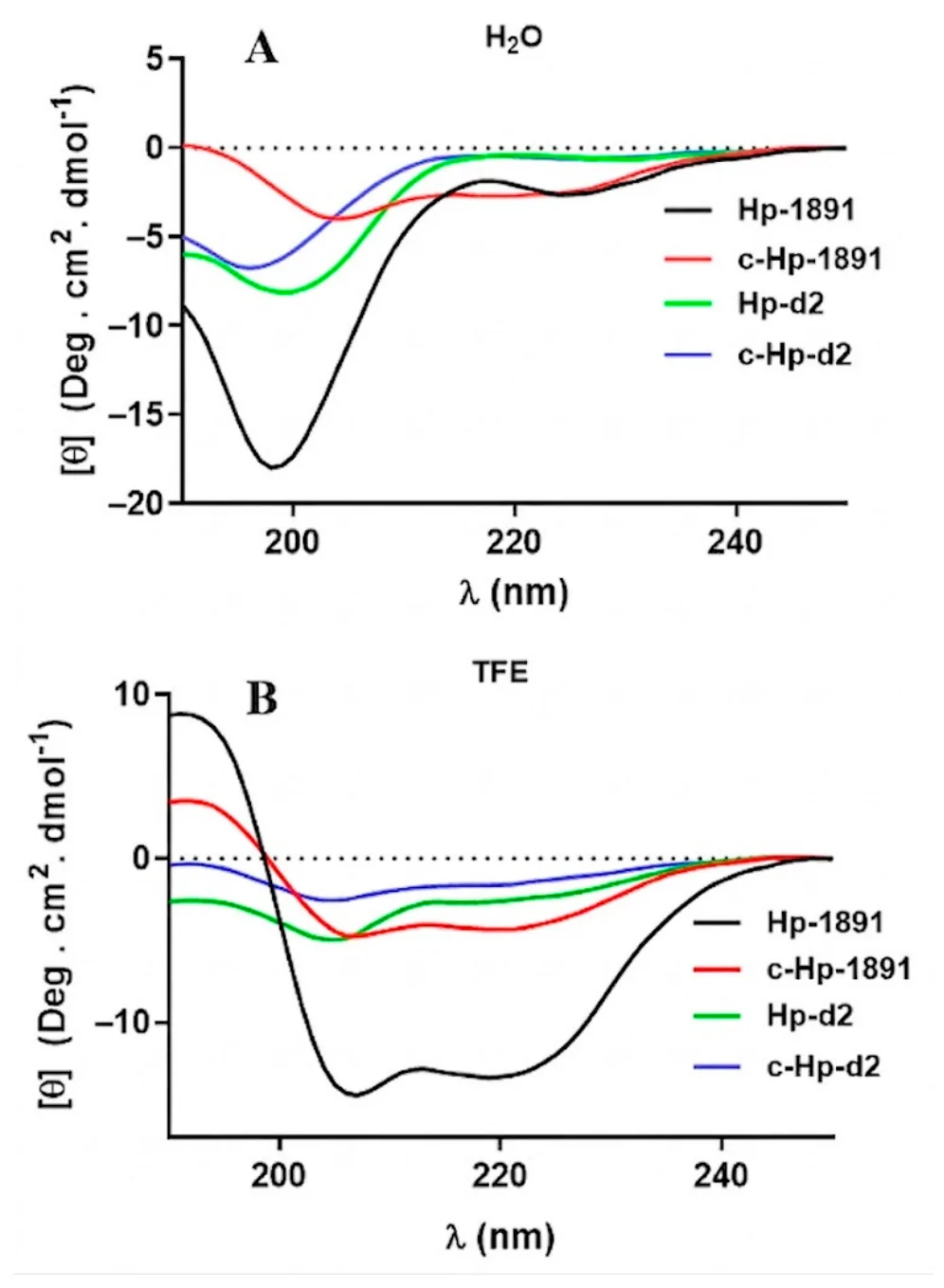
CD spectra of Hp-1891 analogues obtained under the following experimental conditions: (**A**) Milli-Q water and (**B**) TFE/H_2_O (50% *v*/*v*).

To experimentally assess the structural effects of peptide engineering, the secondary structures of selected analogues were analyzed by circular dichroism spectroscopy. Because the objective of this work was to evaluate the effects of D-amino acid substitution and cyclization, four representative peptides were selected for structural characterization: the native linear peptide (Hp-1891), its cyclic counterpart (c-Hp-1891), the most biologically active D-substituted analogue (Hp-d2), and its cyclic derivative (c-Hp-d2). Circular dichroism (CD) spectra were recorded in water and in 50% trifluoroethanol (TFE) (Figure 10).

In aqueous solution, the linear peptides Hp-1891 and Hp-d2 exhibited spectra characteristic of predominantly unstructured conformations. In contrast, the cyclic analogue c-Hp-1891 retained a more structured α-helical conformation, indicating that head-to-tail cyclization without D-substitution increases conformational stability even in a highly polar environment. Upon addition of TFE, both native peptides (Hp-1891 and c-Hp-1891) adopted a characteristic α-helical conformation, displaying the typical double minima at approximately 208 and 222 nm. In contrast, the D-substituted analogues showed a less pronounced helical signature, indicating partial disruption of helix formation. These observations are consistent with previous reports showing that the incorporation of D-amino acids locally disturbs α-helical structures by introducing stereochemical constraints into the peptide backbone [41].

Correlation of the CD profiles with the biological data suggests that conformational organization may contribute to the inhibitory activity of the cyclic analogues. The increased structural organization observed for c-Hp-1891 was associated with enhanced inhibition of AChE and BChE compared with the linear native peptide. However, the high inhibitory activity of c-Hp-d2 despite its lower α-helical propensity indicates that enzyme inhibition cannot be explained solely by the overall helical content. Instead, the specific conformational changes introduced by cyclization and D-amino acid substitution may generate distinct structural arrangements that differentially influence target recognition and interactions with specific residues within the enzymes. This observation was explored through the molecular modeling and molecular dynamics analyses presented previously (see Figure 4, Figure 5 and Figure 7).

Overall, the CD data indicate that cyclization favors structural organization, whereas D-amino acid substitution reduces α-helical propensity, supporting the structural basis for the distinct biological profiles observed among the Hp-1891 analogues.

### 2.11. Structure–Activity Relationships and General Implications of Peptide Engineering

The results obtained in this study show that the biological profile of Hp-1891 can be rationally modulated through complementary peptide engineering strategies. Head-to-tail cyclization emerged as the principal determinant of enzyme inhibition, consistently enhancing the inhibitory activities against AChE, BChE, and SARS-CoV-2 Mpro, whereas D-amino acid substitution exerted a greater influence on membrane selectivity and antioxidant activity. These observations indicate that conformational restriction and stereochemical inversion play distinct but complementary roles in defining peptide function.

The structural analyses support these biological findings. Circular dichroism experiments showed that head-to-tail cyclization favored structural organization, whereas D-amino acid substitution reduced α-helical propensity, in agreement with previous reports describing the helix-disrupting effect of D-residues. Together with the molecular modeling studies, these results suggest that the biological improvements achieved after cyclization arise primarily from conformational preorganization, facilitating more favorable interactions with different molecular targets. The MD-refined complexes extend this interpretation by showing that the same conformational constraint is exploited differently by each enzyme: peripheral-site organization in AChE, deeper gorge accommodation in BChE, and broader occupation of the substrate-recognition cleft in Mpro. Thus, the activity gain cannot be reduced to a single descriptor such as helicity or compactness, but instead reflects target-dependent presentation of the constrained Hp-1891 surface.

Collectively, these findings establish rational peptide engineering as an effective strategy for expanding the functional profile of naturally occurring amphibian peptides and provide a framework for the future optimization of multifunctional peptide scaffolds.

## 3. Materials and Methods

### 3.1. Peptide Synthesis

Linear peptides were synthesized by Fmoc solid-phase peptide synthesis (SPPS) as C-terminal amides using a Rink amide methylbenzhydrylamine (MBHA) resin (Advanced Chemtech, Louisville, KY, USA). Couplings were performed using diisopropylethylamine (DIEA), N-[(1H-benzotriazole-1-yl)(dimethylamino)methylene]-N-methylmethanaminium hexafluorophosphate N-oxide (HBTU) and 1-hydroxy-7-azabenzotriazole (HOAT) as activating agents. Fmoc removal was achieved with 20% piperidine in dimethylformamide (DMF) (*v*/*v*). D-Leu residues were incorporated at the designated positions using Fmoc-D-Leu-OH under the same coupling protocol used for the L-amino acids, with double coupling applied to minimize incomplete reactions at the substituted positions. Final cleavage from the resin and global side-chain deprotection were achieved with a mixture of trifluoroacetic acid (TFA)/H_2_O/triisopropylsilane (TIS) (95:2.5:2.5 *v*/*v*). After 3 h, the resin was filtered off, and the crude peptide was precipitated in cold, dry diethyl ether, centrifuged, and washed several times with cold diethyl ether to remove the scavengers. The product was then lyophilized twice.

Head-to-tail cyclic analogues were synthesized from linear C-terminal carboxylic acid precursors using a 2-chlorotrityl chloride resin (Fluka Chemie AG; USA) and the aforementioned SPPS protocols. After cleaving the fully protected linear peptides from the resin, macrolactamization was carried out in DMF under high-dilution conditions with activating agents to minimize oligomerization. Global side-chain deprotection was subsequently performed using the previously described TFA cleavage cocktail. Successful ring closure was confirmed by a characteristic mass decrease of 17 Da relative to the linear analogues and a marked increase in RP-HPLC retention time.

All synthetic peptides (linear and cyclic) were analysed and purified by reverse-phase HPLC using a C_18_ Jupiter Proteo Phenomenex semi-preparative column (10 µm, 90 Å, 250 × 10 mm) for linear analogues and C4 Jupiter Phenomenex (5 µm, 300 Å, 250 × 21.2 mm) for cyclic, with a gradient of 5–80% acetonitrile (ACN)in water containing 0.1% TFA. MALDI-TOF mass spectrometry was applied to confirm identity and purity (>95%) prior to biological evaluation [40].

### 3.2. Circular Dichroism (CD) Analyses

Far-UV CD measurements were taken on a Jasco J-810 CD spectrometer (JASCO, Tokyo, Japan) in a 0.1-cm path quartz cuvette (Hellma, Mullheim, Germany) and recorded after the accumulation of five runs. CD analyses were recorded in the presence of Trifluoroethanol (TFE) [50% TFE (*v*/*v*)] and Water [42,43]. In all samples, the peptide concentration was 0.2 mg/mL.

### 3.3. Cholinesterase Inhibition Assay

The cholinesterase inhibition was determined following Ellman’s method with modifications [44]. Briefly, increasing concentrations of the peptide were prepared in phosphate buffer (pH 7.5). For AChE, 50 μL of extract was added to a 96 wells microplate followed by the addition of 50 μL of *electric eel* AChE (eeAChE) (0.25 U/mL) in phosphate buffer (pH 7.5). After 30 min of incubation at 25 °C, 100 μL of a solution of 5,5′-dithiobis (2-nitrobenzoic acid) (DTNB) (0.2 mM) and acetylthiocholine iodide (ATCI) (0.24 mM) was added. The absorbance was measured at 405 nm by a microplate reader Thermo Scientific FC. The same procedures were applied for the BChE assay, with modification of the enzyme and substrate used, which were human BChE and S-butyrylthiocholine iodide (BTCI) (7 mM), respectively. In both cases, Rivastigmine was used as a positive control. Percentage of inhibition was calculated as (1 − EAS/EAB) × 100, where EAS and EAB represent the absorbance of enzymatic activity of the sample and basal, respectively. Rivastigmine was used as positive control [27].

### 3.4. Mpro Enzyme Inhibition Assay

The inhibition of Mpro was assessed through a colorimetric final point assay employing the labeled substrate Thr-Ser-Ala-Val-Leu-Gln- para-nitroaniline (pNA) (Sigma-Aldrich, New York, NY, USA). This specific peptide serves as a substrate for the SARS-CoV-2 Mpro. The proteolytic cleavage by the 3CL protease results in the release of the free pNA group from the substrate peptide, with a maximum absorption peak observed at 405 nm. Briefly, 25 µL of increasing concentrations of peptides in 4-(2-hydroxyethyl)-1-piperazineethanesulfonic acid (HEPES)buffer pH 7.0 were mixed with 25 µL of enzyme Mpro (8 µg/mL) (Sigma-Aldrich). The mixture was incubated for 30 min at 35 °C. Following, 50 µL of Mpro substrate (200 µg/mL) was added and incubated for 60 min at room temperature in the dark [14]. Quercetin was used as a positive control [45]. The absorbance was measured at 405 nm. The percentage of inhibition was calculated as (1-EAS/EAB) × 100, where EAS and EAB represent the absorbance enzymatic activity of the sample and basal, respectively.

### 3.5. DPPH Radical Scavenging Activity

The antioxidant activity was determined by 2,2′-diphenyl-1-picrylhydrazyl (DPPH) radical scavenging assay according to the method of Memarpoor-Yazdi with minor modifications [46]. 0.4 mL of increasing concentrations of the peptide were mixed with 1.2 mL of methanol and 0.4 mL of DPPH (0.15 mM in methanol). The mixture was incubated for 30 min in the dark. Ascorbic acid was used as positive control. The absorbance of the resulting solution was measured at 517 nm in a microplate reader Thermo Scientific FC. Percentage of DPPH radical scavenging activity (RSA) was calculated as (1 − As/Ab) × 100, As and Ab representing the absorbance of the sample and basal, respectively.

### 3.6. Hemolytic Activity

The hemolytic activity was determined by previously reported methodology [12]. Briefly, human erythrocytes were isolated from heparinized blood by centrifugation (1000× *g* for 10 min) after washing three times with saline solution. Erythrocytes solutions were prepared to a concentration of 0.4% (*V*/*V*) in isotonic saline solution. Test tubes containing 200 μL of erythrocytes solution were incubated at 37 °C for 60 min with increasing concentrations of the peptide. After centrifugation at 1000× *g* for 5 min, the supernatant absorbance was measured at 405 nm. Lysis induced by 1% Triton X-100 was taken as 100%.

### 3.7. Physicochemical Descriptors and Developability Predictions

The analyzed series comprised native Hp-1891 (RLGTALPALLKTLLAGLNG-NH_2_), five C-terminally amidated linear D-Leu analogues and the corresponding head-to-tail cyclic peptides. GRAVY was calculated as the mean Kyte-Doolittle hydropathy value [15], the hydrophobic fraction from the proportion of hydrophobic residues, the aliphatic index from Ala/Val/Ile/Leu content [16], and aromaticity from the Phe/Trp/Tyr fraction. Net charge at pH 7.4 was estimated with the Henderson-Hasselbalch equation using pKa values of 10.67 for Lys, 12.10 for Arg and 9.38 for the N terminus; the C-terminal amide was treated as neutral and terminal groups were absent in cyclic peptides, following the general approach used in modlAMP [47]. Topology-aware molecular weight, TPSA, HBD/HBA counts, the standard RDKit rotatable-bond descriptor, and Wildman-Crippen MolLogP were calculated from stereospecific neutral covalent SMILES with RDKit (https://zenodo.org/records/21741729, accessed on 28 July 2026). In this topological rotatable-bond descriptor, bonds that are members of rings are excluded; therefore, cyclization can produce a large numerical decrease without implying that the macrocycle is conformationally rigid. TPSA and MolLogP used the established methods [48,49]. Experimental RP-HPLC retention times were taken from the analytical characterization described above.

Developability-related properties were predicted with PeptiVerse using the SMILES input mode so that D/L stereochemistry, the C-terminal amide of linear peptides and head-to-tail cyclic connectivity were explicitly retained [18]. In the ionized SMILES, the N terminus, Lys and Arg were protonated in linear peptides while the C-terminal amide was neutral, giving a formal charge of +3; cyclic peptides lacked free termini and had a formal charge of +2 from Lys and Arg. Penetrance, peptide half-life and PAMPA permeability were analyzed, with half-life reported in hours and PAMPA as log Peff. Native linear Hp-1891 was used as the reference for all comparisons.

### 3.8. Peptide Structure Modeling and Conformational Selection

3D structures of Hp-1891 and its analogues were generated in linear and head-to-tail cyclic forms using Rosetta release 371. Linear peptides were modeled de novo with a fragment-free full-atom RosettaScripts protocol [50]. Peptide chains were constructed with PeptideStubMover, backbone dihedral angles were randomized according to residue-specific RamaPrePro distributions, and conformations were relaxed with the ref2015 energy function [51]. Sampling comprised 3000 Monte Carlo trials combining Small and Shear backbone moves with equal probability, a maximum perturbation of 25 degrees and a Monte Carlo temperature of 2.0, followed by two FastRelax cycles. One hundred independent structures were generated for each linear peptide.

Head-to-tail cyclic peptides were modeled with Rosetta simple_cycpep_predict using generalized kinematic closure between the C-terminal carbonyl carbon and N-terminal nitrogen. This Rosetta framework supports mixed L/D stereochemistry and backbone-cyclized peptide sampling [52,53]. Calculations were performed in batches of 1000 attempted predictions and repeated until at least 100 successfully closed conformations were obtained for each sequence. D-Leu stereochemistry was explicitly defined at substituted positions. Linear and cyclic ensembles were clustered with Rosetta energy_based_clustering using backbone Cartesian RMSD and a 2.0 Å clustering radius, with cyclic topology considered for macrocycles; the center of the most populated cluster was selected for MD refinement, with ties resolved by Rosetta energy [53].

### 3.9. Molecular Dynamics Refinement of Isolated Peptides

The Rosetta-selected conformation of each peptide was refined by 100 ns of all-atom MD in explicit water using AMBER 22. Systems were prepared with tLEaP, described with ff14SB [54], solvated with TIP3P water in a periodic box extending at least 12 Å from the solute [55], and neutralized with counterions. D-Leu residues were represented with force-field-compatible D-amino-acid parameters while preserving inverted Cα stereochemistry; linear peptides retained the C-terminal amide and cyclic peptides retained the head-to-tail amide bond. After solvent-restrained and whole-system minimization, systems were heated to 298 K, equilibrated for 200 ps and simulated for 100 ns in the isothermal-isobaric (NPT) ensemble at 298 K and 1 atm with PMEMD on GPU hardware, using a 2 fs time step and coordinates saved every 1 ps. AMBER/tLEaP and PMEMD are described in the AMBER program literature [56], and the GPU implementation for explicit-solvent PME simulations is described in Salomon-Ferrer 2013 [57]. The protocol follows our previous peptide MD workflows [14,25,26].

### 3.10. Peptide-Enzyme Docking and Molecular Dynamics Simulations

Representative aqueous peptide conformations were docked semi-flexibly with TcAChE (PDB 1FSS [27]), human BChE (PDB 2PM8 [58]) and SARS-CoV-2 Mpro (PDB 6LU7 [28]) using the HADDOCK2.4 web server (https://wenmr.science.uu.nl/haddock2.4/). Crystallographic ligands, solvent and non-required ions were removed, and fasciculin was removed from 1FSS. The peptide was treated as the flexible partner and receptor restraints encompassed the relevant cholinesterase gorge/peripheral regions or the Mpro substrate-binding cleft. Complexes were clustered and ranked by the HADDOCK score, and all cluster representatives of each complex were then rescored with PRODIGY [22,23], a contact-based predictor of binding affinity for protein–protein and protein–peptide interfaces that derives ΔG from the number and chemical nature of the inter-residue contacts across the interface and from the composition of the non-interacting surface. PRODIGY contains no van der Waals, electrostatic, desolvation or restraint term and therefore shares neither functional form nor parameters with the HADDOCK score, so agreement between the two rankings is informative rather than circular. In all six complexes the HADDOCK-selected pose fell within the five most favourable poses of the independent ranking (Table S1) and was promoted to MD refinement without modification. Each complex was then solvated and simulated with AMBER/ff14SB/TIP3P using the same minimization, heating, 200 ps equilibration and 100 ns NPT production protocol described above, allowing the docking pose to relax in explicit solvent without docking restraints. To avoid basing the analysis on a single trajectory, every complex was simulated in three fully independent 100 ns replicates started from the same equilibrated structure with independently generated initial velocities.

### 3.11. Trajectory Analysis, Representative Structures and Interaction Occupancy

Trajectory analysis was performed with ShineMD [24]. Root-mean-square deviations were computed over the full 100 ns of each replicate after mass-weighted superposition of the receptor backbone onto the energy-minimised starting structure of that replicate; the peptide heavy-atom RMSD was measured in the receptor-superposed frame and therefore reports the combined conformational and positional drift of the ligand within the binding site. Representative structures and contact occupancies were derived from the last 20 ns of each of the three replicates of a complex after alignment to the enzyme. RMSD-based clustering was performed on this pooled ensemble, and the medoid of the most populated cluster was exported as the representative structure, so that the structure shown for each complex is supported by all three trajectories. Receptor-peptide residue contact maps were calculated over the same pooled ensemble using a 4.5 Å heavy-atom contact cutoff; occupancy was reported as the percentage of the analyzed frames in which each residue pair formed at least one contact.

Peptide RMSD descriptors were computed per replicate over the 50–100 ns window, so that the initial relaxation of the docked pose is excluded. The nine cyclic replicates (three complexes × three replicates) were compared with the nine linear replicates by two-sided Mann–Whitney U tests, with Cliff’s delta as effect size; because replicates of the same complex are not fully independent observations, the same comparison was also performed after averaging the three replicates of each complex (n = 3 targets), which reproduced the direction of every effect. Statistical analysis was performed in Python 3 with SciPy (v 1.11).

### 3.12. Peptide Burial and Insertion Analysis

Peptide burial was estimated from solvent-accessible surface area (SASA) [58] by comparing the peptide in the complex with the isolated peptide in the identical bound conformation; the buried fraction was normalized to the isolated-peptide SASA. Cholinesterase insertion was additionally measured by projecting the peptide heavy-atom centroid along a functional gorge axis: Trp279 to catalytic Ser200 for TcAChE and Tyr332 to catalytic Ser198 for hBChE. Trp279 and Tyr332 represent peripheral entrance/recognition positions, whereas Ser200 and Ser198 are the corresponding catalytic nucleophiles, making these axes entrance-to-catalytic-center measures. Because Mpro contains an open cleft rather than an elongated gorge, insertion was quantified relative to the midpoint of the His41-Cys145 catalytic dyad as the fraction of peptide heavy atoms and the number of peptide residues within 10 Å of that center.

### 3.13. Statistical Analysis

All biological assays were performed in triplicate (n = 3). Data are expressed as mean ± SEM. Statistically significant differences among groups were assessed by one-way ANOVA followed by Tukey’s HSD post hoc test (α = 0.05) [59,60]. All analyses were performed using Python (v3.11) with the SciPy (v1.11) and NumPy (v1.26) libraries. Statistically distinct groups are indicated in figures using compact letter display (CLD) notation; bars sharing the same letter are not significantly different (*p* > 0.05).

## 4. Conclusions

This work shows that Hp-1891 is a versatile molecular scaffold for rational peptide engineering. A focused library of twelve analogues generated through site-specific D-amino acid substitution and head-to-tail cyclization revealed that both strategies exert complementary effects on peptide function. Whereas cyclization enhanced inhibition of AChE, BChE, and SARS-CoV-2 Mpro, D-amino acid incorporation primarily modulated antioxidant activity, secondary structure, and toxicity. Together, these findings establish rational peptide engineering as an effective approach for transforming naturally occurring amphibian peptides into multifunctional scaffolds and provide useful design principles for the development of next-generation peptide therapeutics.

## Figures and Tables

**Table 1:** Experimental RP-HPLC retention time, selected molecular descriptors and predicted properties for Hp-1891 and its analogues.

Peptide	Modification	HPLC Rt (Min) *	Net Charge (pH 7.4)	MW (Da)	TPSA (Å^2^)	HBD/HBA	Rotatable Bonds	MolLogP	Penetrance	PAMPA (log Peff)
Hp-1891	Native linear, −CONH_2_	6.24	+2.989	1891.339	755.59	26/25	62	−6.663	0.687	−12.702
Hp-d1	*l*_14_	4.71								
Hp-d2	*l*_9_, *l*_14_	5.54								
Hp-d3	*l*_9_, *l*_13_, *l*_14_	4.63								
Hp-d4	*l*_9_, *l*_10_, *l*_13_	4.94								
Hp-d5	*l*_9_, *l*_10_, *l*_13_, *l*_14_	4.77							0.658	−13.276
c-Hp-1891	Native cyclic	23.60	+1.999	1874.308	715.58	25/24	26	−5.977	0.775	−10.354
c-Hp-d1	c(*l*_14_)	22.88								
c-Hp-d2	c(*l*_9_, *l*_14_)	22.23								
c-Hp-d3	c(*l*_9_, *l*_13_, *l*_14_)	21.51								
c-Hp-d4	c(*l*_9_, *l*_10_, *l*_13_)	21.59								
c-Hp-d5	c(*l*_9_, *l*_10_, *l*_13_, *l*_14_)	20.75								

GRAVY (0.837), hydrophobic-residue fraction (0.526), aliphatic index (159.5), and aromaticity (0.000) were identical for all twelve peptides because L→l inversion and head-to-tail closure do not change side-chain identities. HPLC retention times and PeptiVerse predictions are reported individually. Descriptor values that are identical for all six linear peptides or all six cyclic peptides are shown once in vertically merged cells to avoid repetition. The RDKit rotatable-bond value is a formal two-dimensional descriptor: ring bonds are not counted as rotatable, so macrocyclization converts many backbone bonds that contribute in the linear peptide into ring bonds. The 62-to-26 decrease therefore reflects the descriptor definition and should not be interpreted as a proportional loss of conformational mobility. * See RP-HPLC chromatograms in Figure S1—Supplementary Materials.

**Table 2:** AChE and BChE Inhibitory activity expressed.

Analogues	IC_50_ (µM)	
	AChE	BChE
c-Hp-1891	91.54 ± 2.30	92.52 ± 3.42
c-Hp-d1	-	45.48 ± 0.80
c-Hp-d2	310.6 ± 7.45	54.37 ± 1.10
c-Hp-d3	-	113.8 ± 3.13
c-Hp-d4	-	49.29 ± 1.55
c-Hp-d5	-	149.1 ± 8.02
Rivastigmine (C+)	33.17 ± 6.00	0.08 ± 0.00

C+: positive control. All values were determined by regression analyses of three replicates and expressed with a confidence of 95%.

**Table 3:** Mpro **IC_50_** activity.

Analogues	IC_50_ (μM)
c-Hp-1891	402.5 ± 7.2
c-Hp-d2	332.7 ± 4.0
c-Hp-d3	398.2 ± 1.1
c-Hp-d5	720.5 ± 6.3
Quercetin (C+)	25.1 ± 7.1

C+: positive control. All values were determined by regression analyses of three replicates and expressed with a confidence level of 95%.

## Data Availability

The raw data supporting the conclusions of this article will be made available by the authors on request.

## Supplementary Materials

The following supporting information can be downloaded at: https://www.mdpi.com/article/10.3390/molecules31183240/s1, Figure S1. RP-HPLC profiles of the Hp-1891 analogues. Figure S2. AChE and BChE inhibition dose–response curves of Hp-1891 and its engineered analogues. Figure S3. Mpro inhibition dose–response curves of Hp-1891 and its engineered analogues. Table S1. Independent rescoring of the HADDOCK binding modes with PRODIGY. Figure S4. Receptor backbone RMSD. Figure S5. Peptide RMSD.
