# Supplementary material for: Head-to-Tail Cyclization and D-Amino Acid Substitution Redesign the Biological Activities of a Naturally Occurring Amphibian Peptide
Source: Molecules. 2026 Sep 14;31(18):3240. doi: 10.3390/molecules31183240 (PMC13609225; doi:10.3390/molecules31183240)
Supplement: Supplementary file 1 [file molecules-31-03240-s001.zip › molecules-4547001-supplementary.pdf]

SUPPLEMENTARY MATERIAL

Head-to-Tail Cyclization and D-Amino Acid Substitution Redesign the Biological Activities of a Naturally Occurring Amphibian Peptide

Húmpola María Verónica[1] (†), Spinelli Roque[1,2] (†), Sanchís Ivan[1,2], deOrellana Milagros[1,2], Albericio Fernando*[3,4], Siano Álvaro*[1,2]

1. Laboratorio de Peptidos Bioactivos, Department of Organic Chemistry, Faculty of Biochemistry and Biological Sciences, National University of the Littoral, Ciudad Universitaria UNL, 3000 Santa Fe, Argentina.

2. National Scientific and Technical Research Council (CONICET), Ministry of Science, Technology and Innovation, Godoy Cruz 2290, Ciudad de Buenos Aires, Argentina.

3. School of Chemistry and Physics, University of KwaZulu-Natal, Durban 4001, South Africa.

4. Department of Inorganic and Organic Chemistry, University of Barcelona, 08028 Barcelona, Spain.

(†) These authors contributed equally to this work

* Correspondence: albericio@ukzn.ac.za, asiano@fbcb.unl.edu.ar

ABSTRACT

Peptide engineering has emerged as a powerful strategy to optimize naturally occurring peptides. Here, the amphibian skin peptide Hp-1891 from *Boana pulchella* was selected as a model scaffold to investigate the effects of two complementary engineering approaches, namely site-specific D-amino acid substitution and head-to-tail cyclization. A library of twelve analogues was synthesized by Fmoc-based solid-phase peptide synthesis and evaluated for inhibitory activity against acetylcholinesterase (AChE), butyrylcholinesterase (BChE), and the SARS-CoV-2 main protease (Mpro), together with antioxidant and hemolytic activities. Circular dichroism spectroscopy and molecular modeling were performed to investigate the structural basis of the observed biological effects. Head-to-tail

cyclization consistently enhanced inhibition of AChE, BChE, and Mpro, whereas D-amino acid substitution exerted a greater influence on antioxidant activity and hemolysis. Among the analogue library, c-Hp-d2 emerged as the most promising multifunctional peptide, displaying enhanced inhibition of all three enzymes while maintaining reduced hemolytic activity compared with the native peptide. Structural analyses indicated that cyclization promoted conformational organization, whereas D-amino acid incorporation reduced α-helical propensity. These findings demonstrate that rational peptide engineering effectively reshapes the biological profile of amphibian peptides and highlight head-to-tail cyclization as a versatile strategy for generating multifunctional peptide scaffolds with therapeutic potential.

KEYWORDS

Amphibian peptides, cyclic peptide, D-amino acids, Cholinesterase inhibitors, SARS-CoV-2, Main Protease inhibitors.

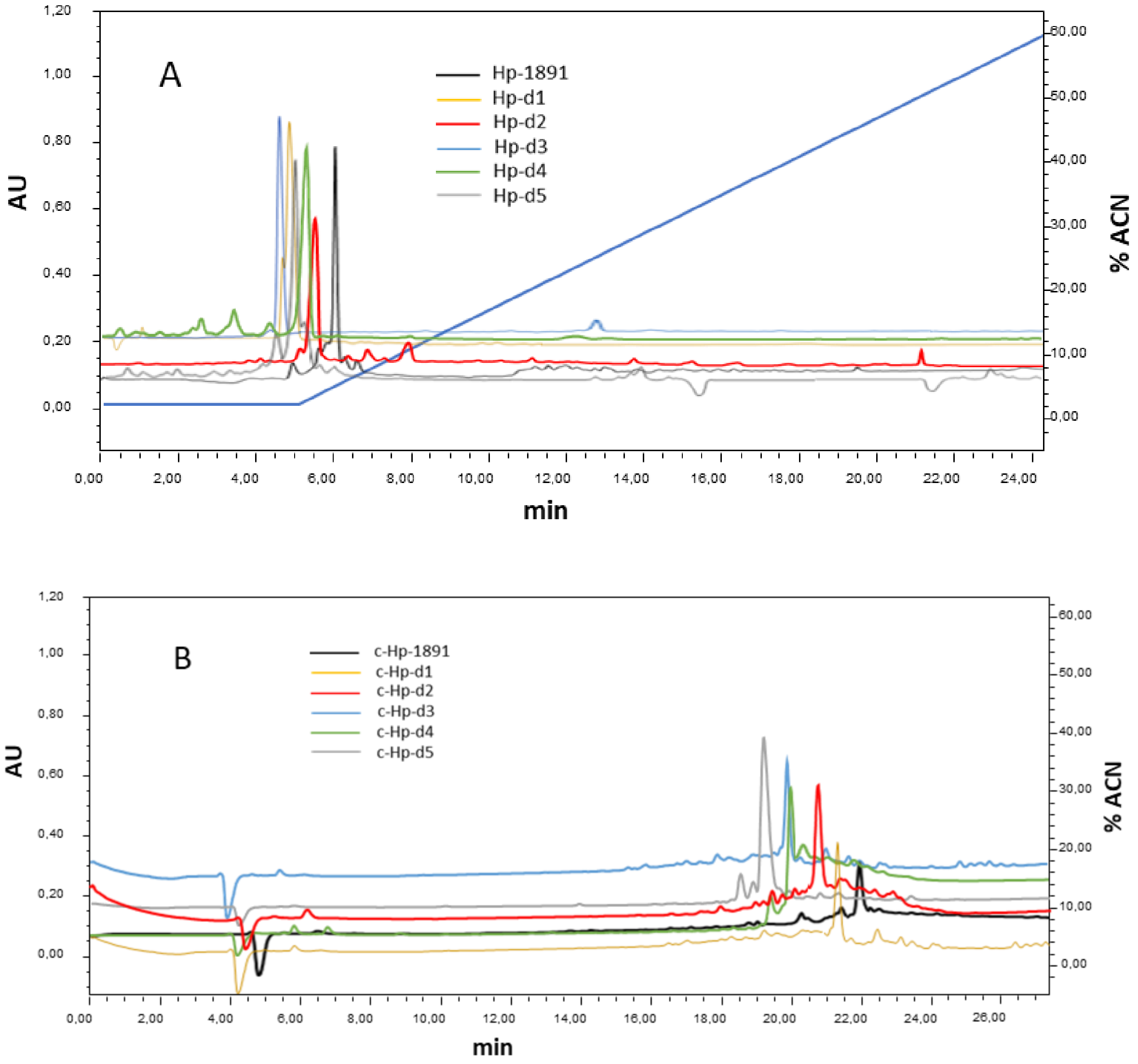

Figure S1. RP-HPLC profiles of the Hp-1891 analogues. A linear gradient from 5 to 80% of solution B was applied, using solution A (0.1% TFA in water) and solution B (0.1% TFA in acetonitrile). Detection was performed at 220 nm. (A) Linear analogues analyzed using a C18 column. (B) Cyclic analogues analyzed using a C4 column.

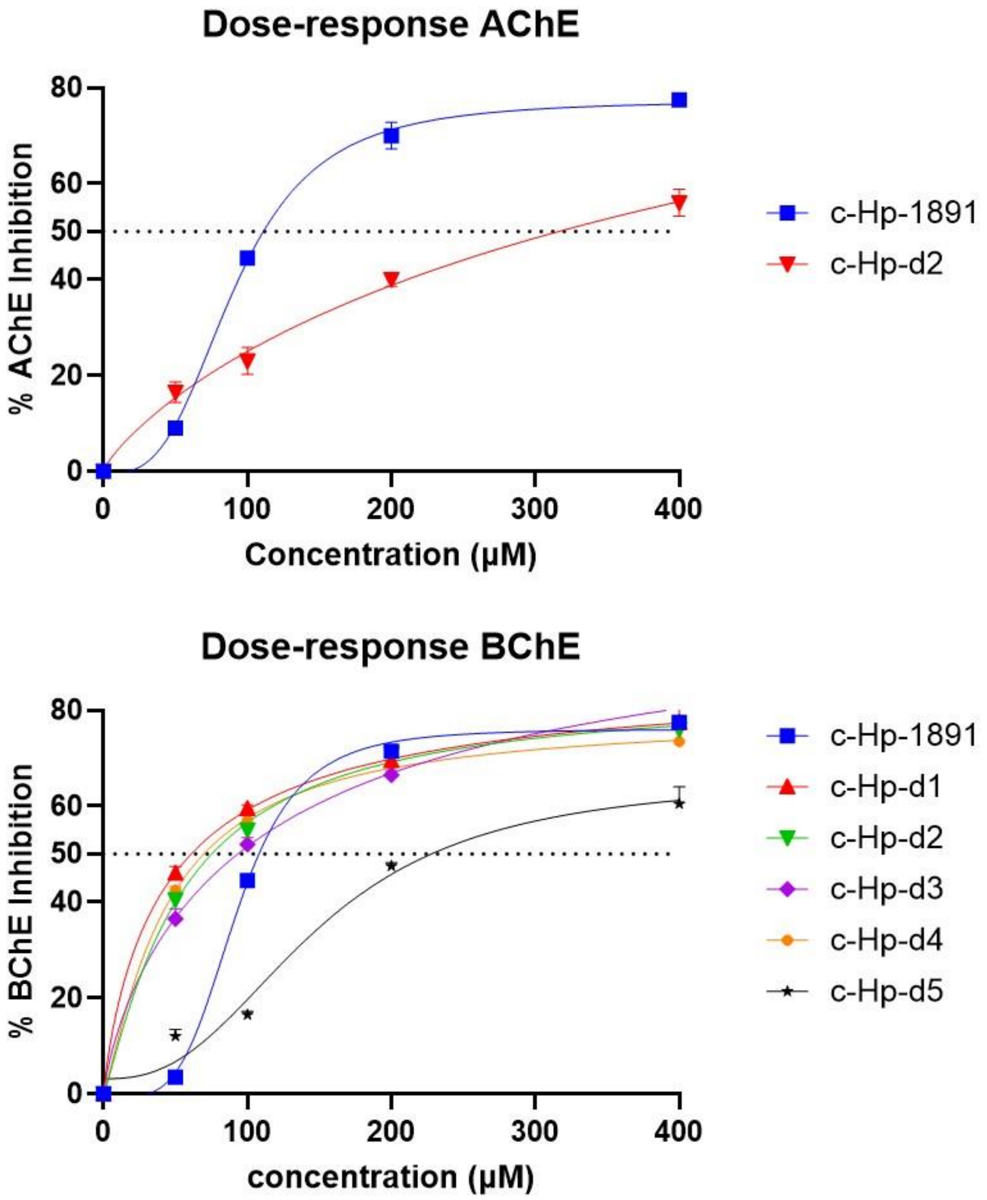

Figure S2. AChE and BChE inhibition dose–response curves of Hp-1891 and its engineered analogues.

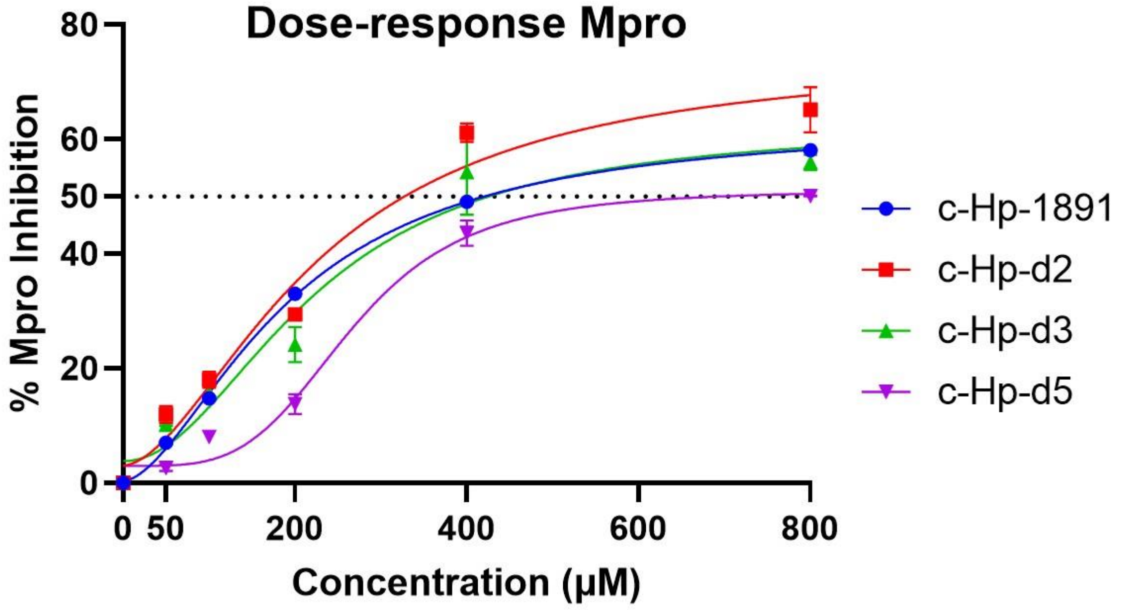

Figure S3. Mpro inhibition dose–response curves of Hp-1891 and its engineered analogues.

Table S1. Independent rescoring of the HADDOCK binding modes with PRODIGY. All cluster representatives generated by HADDOCK for each peptide–enzyme complex were rescored with PRODIGY and re-ranked by the predicted binding free energy. The rank is the position of the pose promoted to molecular dynamics within that independent ranking (rank 1 = most favourable). In all six complexes the HADDOCK-selected pose falls within the five most favourable poses of the PRODIGY ranking.

Complex	HADDOCK score of the selected cluster	Pose promoted to MD	PRODIGY ΔG (kcal mol^{-1})	PRODIGY rank
c-Hp-1891 / TcAChE	−88.5	cluster3_1	−11.12	3
Hp-1891 linear / TcAChE	−80.9	cluster4_1	−10.64	1
c-Hp-d1 / hBChE	−104.8	cluster3_1	−12.90	4
Hp-1891 linear / hBChE	−79.0	cluster1_1	−11.75	1
c-Hp-d2 / Mpro	−63.8	cluster5_1	−12.06	5
Hp-1891 linear / Mpro	−76.4	cluster8_1	−14.08	3

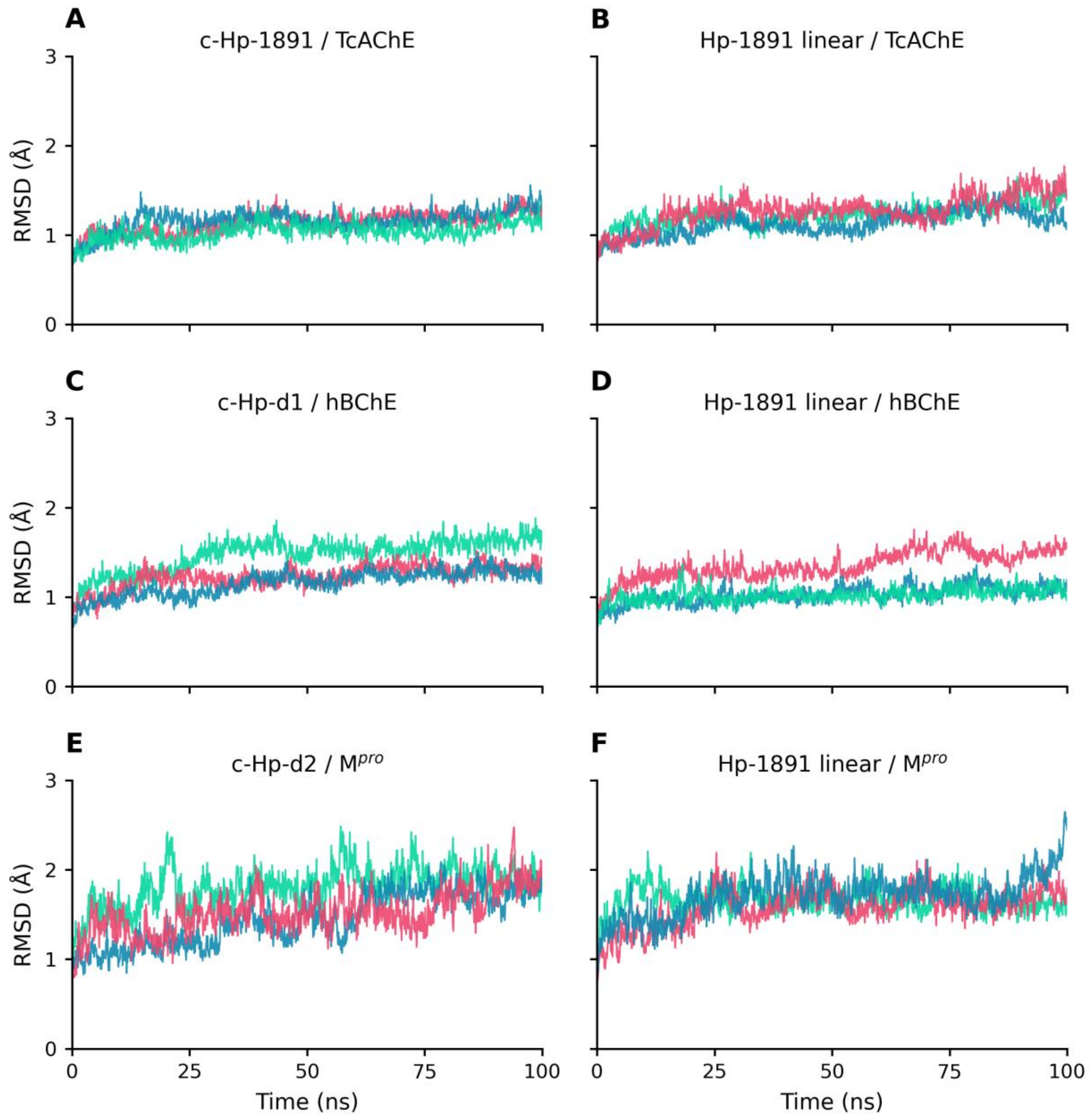

Figure S4. Receptor backbone RMSD. Time evolution of the receptor backbone root-mean-square deviation (RMSD, Å) during three independent 100 ns molecular dynamics replicates of each peptide–target complex: (A) c-Hp-1891/TcAChE, (B) Hp-1891 linear/TcAChE, (C) c-Hp-d1/hBChE, (D) Hp-1891 linear/hBChE, (E) c-Hp-d2/Mpro and (F) Hp-1891 linear/Mpro. RMSD values were computed against the energy-minimised starting structure of each replicate after mass-weighted superposition of the receptor backbone. The three coloured traces in each panel correspond to the three independent replicates (blue, replicate 1; green, replicate 2; red, replicate 3); replicate colours are not comparable between panels.

All panels share the same vertical scale (0–3 Å). Every receptor remained structurally stable, with RMSD values below 2.7 Å throughout the simulations.

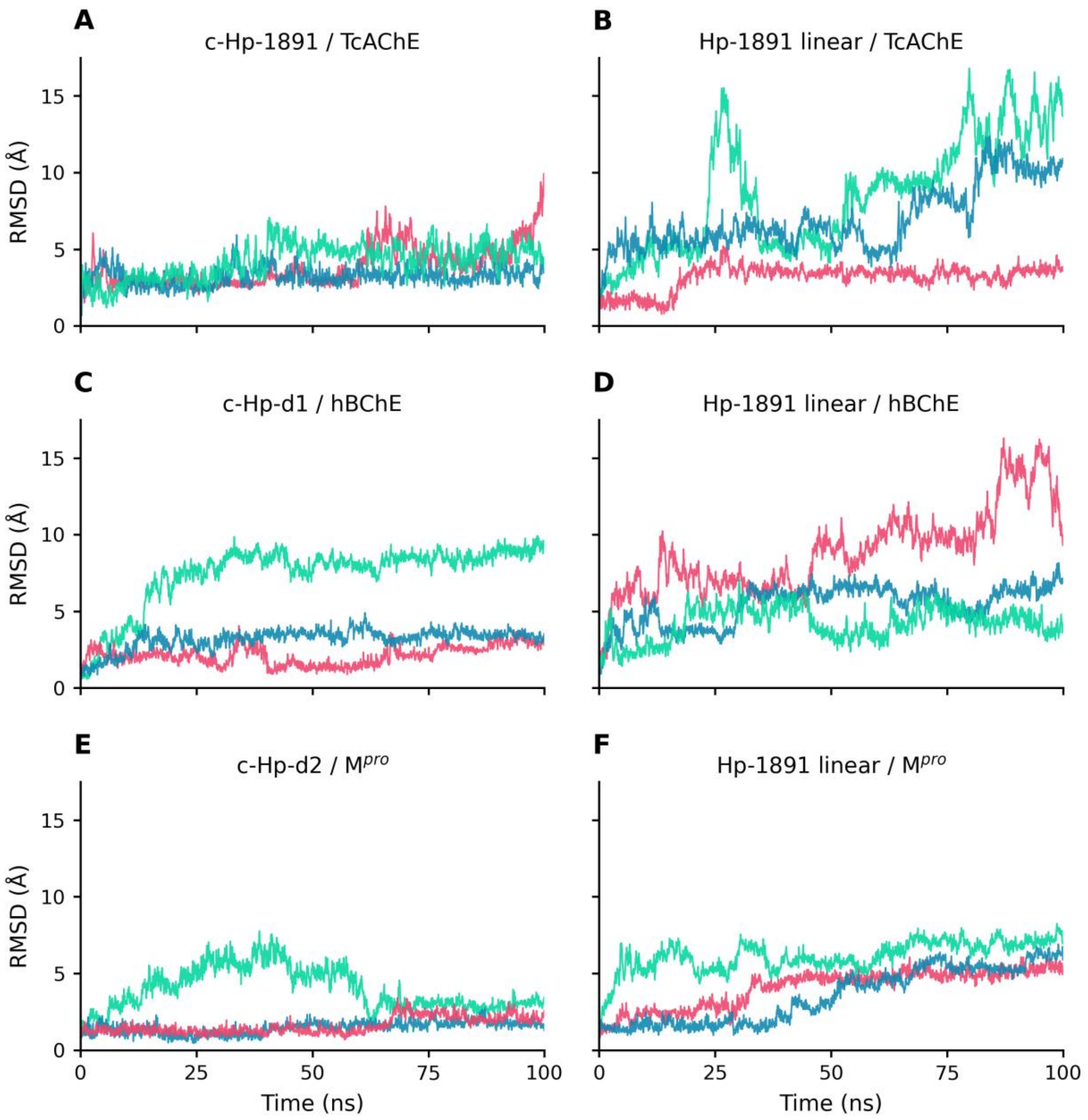

Figure S5. Peptide RMSD. Time evolution of the peptide heavy-atom root-mean-square deviation (RMSD, Å) during the same three independent 100 ns replicates; panel layout as in Figure S4. The peptide RMSD was measured after superposition of the receptor, so it reports the combined conformational and positional drift of the ligand within the binding site. All panels share the same vertical scale (0–17.5 Å). The cyclic analogues (left column) sample a narrower RMSD range than the linear parent peptide (right column), which

undergoes excursions above 10 Å in individual replicates of the TcAChE and hBChE complexes.
